# Engineered atherosclerosis-specific zinc ferrite nanocomplex-based MRI contrast agents

**DOI:** 10.1186/s12951-016-0157-1

**Published:** 2016-01-16

**Authors:** Rajneesh Chaudhary, Kislay Roy, Rupinder Kaur Kanwar, Ken Walder, Jagat Rakesh Kanwar

**Affiliations:** Nanomedicine-Laboratory of Immunology and Molecular Biomedical Research (NLIMBR), School of Medicine (SoM), Faculty of Health, Centre for Molecular and Medical Research (C-MMR), Deakin University, Pigdons Road, Waurn Ponds, Geelong, VIC 3217 Australia; Metabolic Research Unit, School of Medicine (SoM), Faculty of Health, Centre for Molecular and Medical Research (C-MMR), Deakin University, Pigdons Road, Waurn Ponds, Geelong, VIC 3217 Australia

**Keywords:** Atherosclerosis, Zinc doped ferrite nanoparticles, Heat shock protein, Lactoferrin, *Psammomys obesus*, Magnetic resonance imaging

## Abstract

**Background:**

Cardiovascular diseases are the most prevalent cause of morbidity and mortality affecting millions of people globally. The most effective way to counter cardiovascular complications is early diagnosis and the safest non-invasive diagnostic approach is magnetic resonance imaging (MRI). In this study, superparamagnetic ferrite nanoparticles doped with zinc, exhibiting highly enhanced saturation magnetization and T2 and computed tomography (CT) contrast were synthesized. These nanoparticles have been strategically engineered using bovine lactoferrin (Lf), polyethylene glycol (PEG), and heat shock protein (Hsp)-70 antibody specifically targeting atherosclerosis with potential therapeutic value. The nanocomplexes were further validated in vitro to assess their cytotoxicity, internalization efficiency, effects on cellular proliferation and were assessed for MRI as well as X-ray CT in ex vivo *Psammomys obesus* rat model.

**Results:**

Optimized zinc doped ferrite nanoparticles (Zn_0.4_Fe_2.6_O_4_) with enhanced value of maximum saturation magnetization value on 108.4 emu/g and an average diameter of 24 ± 2 nm were successfully synthesized. Successfully incorporation with bovine lactoferrin, PEG and Hsp-70 (70 kDa) antibody led to synthesis of spherical nanocomplexes (size 224.8 nm, PDI 0.398). A significantly higher enhancement in T2 (p < 0.05, 1.22-fold) and slightly higher T1 (1.09-fold) and CT (1.08-fold) contrast compared to commercial ferrite nanoparticles was observed. The nanocomplexes exhibited effective cellular internalization within 2 h in both THP-1 and Jurkat cells. MRI scans of contrast agent injected animal revealed significant arterial narrowing and a significantly higher T2 (p < 0.05, 1.71-fold) contrast in adult animals when compared to juvenile and control animals. The excised heart and aorta agar phantoms exhibited weak MRI contrast enhancement in juvenile animal but significant contrast enhancement in adult animal specifically at the aortic arch, descending thoracic aorta and iliac bifurcation region with X-ray CT scan. Histological investigation of the contrast agent injected aorta and heart confirmed site target-specific accumulation at the atherosclerotic aortic arch and descending thoracic aorta of the adult animal with severely damaged intima full of ruptured microatheromas.

**Conclusion:**

Overall, the study demonstrates the strategic development of nanocomplex based bimodal MRI and CT contrast agents and its validation on *Psammomys obesus* for atherosclerosis diagnostics.

**Electronic supplementary material:**

The online version of this article (doi:10.1186/s12951-016-0157-1) contains supplementary material, which is available to authorized users.

## Background

Atherosclerosis is a root cause of cardiovascular diseases (CVD) leading to morbidity and mortality in millions of people worldwide [1, 2] and is often symptomatically silent until the occurrence of a catastrophic clinical event such as myocardial infarction, stroke or peripheral vascular diseases. The most reliable and conventional invasive technique for the visualization of affected blood vessels, either arteries or veins is catheter angiography [3] which is highly invasive, requires a complicated operative procedure, exposes the patient to X-ray radiation, is time consuming and the catheter may injure the artery which may lead to internal bleeding. On the other hand, magnetic resonance imaging (MRI) is a non-invasive, painless, and safe diagnostic technology that uses a magnetic field and radio frequency to produce a detailed image of the body’s organs and structures with high spatial resolution depending on the differences in longitudinal (T1) and transverse (T2) proton relaxation times of contrast agents that can effectively improve the MRI contrast between normal and diseased tissues [4]. Paramagnetic gadolinium chelates are the most widely used commercial MRI contrast agents used for MRI based diagnostics [5]. However, Gadolinium exhibits high in vivo toxicity and therefore must be modified with organic chelators such as diethylene triamine pentaacetic acid (Gd-DTPA) and 1,4,7,10-tetraazacyclododecane-1,4,7,10-tetraacetic acid (Gd-DOTA) [6]. Even after chelation gadolinium based contrast agents exhibit nephrogenic fibrosing dermopathy [7] and nephrogenic systemic fibrosis [8] in patients with renal insufficiency, primarily due to competitive transmettalation of divalent or trivalent cations with gadolinium [7, 9].

Engineered nanoparticulate contrast agents progressively contribute to the field of cardiovascular diagnostics and molecular imaging by providing several advantages over traditional imaging contrast agents [10, 11]. The most extensively studied class of magnetic nanoparticles for contrast agents development are iron oxide nanoparticles, also referred as ferrite nanoparticles. This is due to their superparamagnetic nature, biodegradability, inoffensive toxicity profile, low particle dimensions, high surface to volume ratio and reactive surface readily modifiable with biocompatible coatings, targeting/therapeutic molecules as well as other imaging modalities [4, 12–15]. The mode of action of ferrite nanoparticles is by enhancing the relaxation rate or shortening the T2 relaxation time by increasing field inhomogeneity of surrounding protons to creating strong negative MRI contrast [4, 12–15]. Ferrite nanoparticles can be doped with zinc, a micronutrient to considerably enhance the saturation magnetization (M_S_) for improved MRI contrast while effectively reducing the chances of iron overloading by iron cation replacement [16, 17]. Zinc is the most suitable candidate as a dopant as Food and Drug Administration (FDA) recommends a high reference daily value (DV) for zinc and iron to be 15 and 18 mg respectively, considerably much more than the values for other possible dopants, for example manganese and cobalt at 2 mg (http://www.fda.gov).

In the present study, we developed and validated highly superparamagnetic zinc doped ferrite (ZF) nanoparticle based nanocomplexes with minimized in vivo toxicity compared to commercial ferrite based MRI contrast agents. Three types of bovine lactoferrin-zinc ferrite (Lf-ZF) nanocomplexes were developed, Hsp-70 targeted lactoferrin embedded ZF nanoparticles (Hsp-70 Lf-ZF), Hsp-70 targeted chitosan encapsulated lactoferrin embedded ZF nanoparticles (Hsp-70 Ch-Lf-ZF) and Hsp-70 targeted polyethylene glycol assisted lactoferrin embedded ZF (Hsp-70 Lf-PEG-ZF). Lf-ZF forms the core of all nanocomplexes, where superparamagnetic ZF nanoparticles generate MRI contrast and lactoferrin acts as a targeting and therapeutic protein against atherosclerosis. Lactoferrin is a naturally available protein with anti-bacterial, anti-viral, anti-inflammatory and anti-carcinogenic properties and also helps in boosting the immune system by activation of T lymphocytes [18, 19]. Since, the temporal expression of Hsps’ has been demonstrated at atherosclerotic lesion [20], this is the first attempt demonstrating Hsp-70 targeted approach for atherosclerosis diagnostics. Validation of the developed nanocomplex contrast agent was performed with Psammomys obesus (*P. obesus*), a polygenic animal model for type-2 diabetes, obesity and atherosclerosis [21, 22].

## Results and discussion

### Characterization of zinc doped ferrite based nanocomplexes

**Fig. 1:**
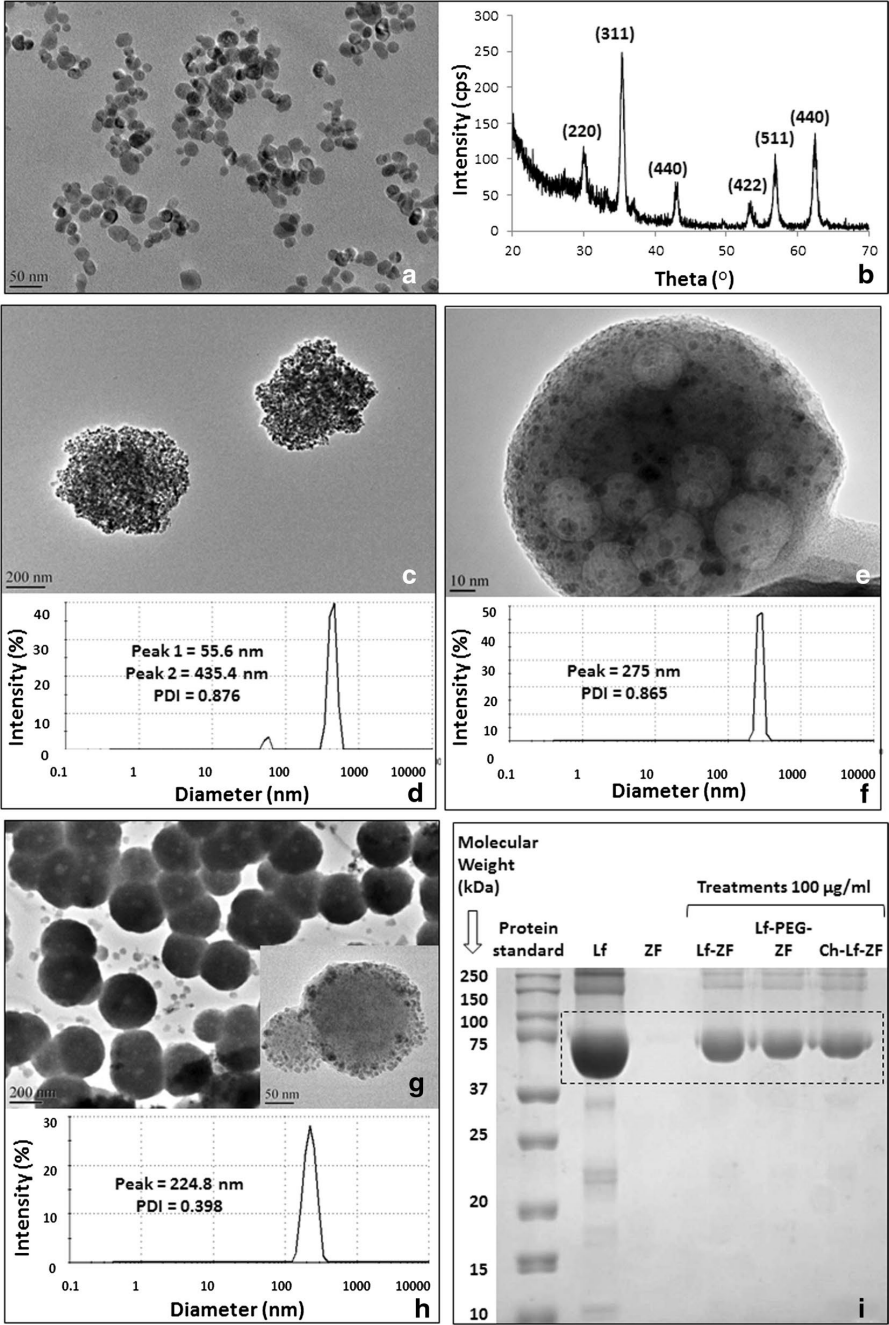
Characterization of zinc doped ferrite nanoparticles and their nanocomplexes. **a **Transmission electron microscopy (TEM) image confirmed formation of uniformly sized spherical zinc-doped ferrite nanoparticles (Zn_0.4_Fe_2.6_O_4_) with an average diameter of 24 ± 2.8 nm. **b** X-ray differactogram revealed the formation of crystallographic spinel structure of zinc-doped ferrite nanoparticles. **c** TEM image revealing the formation of Hsp-70 Lf- ZF nanocomplex. **d** DLS analysis of Hsp-70 Lf-ZF nanoparticles revealed bimodal hydrodynamic size distribution with the prominent primary peak at 435.4 nm and a secondary peak at 55.6 nm with a polydispersity index of 0.876. **e** TEM image revealed the formation of Hsp-70 Ch-Lf-ZF nanocomplex. **f** DLS analysis revealed the hydrodynamic diameter of 275 nm with a PDI of 0.865. **g** TEM image revealing the formation of Hsp-70 Lf-PEG-ZF nanocomplex and a highly magnified image (*inset*). **h** Dynamic light scattering (DLS) revealed the hydrodynamic diameter of 224.8 nm with a polydispersity index of 0.398. **i** Sodium dodecyl sulfate—polyacrylamide gel electrophoresis (SDS-PAGE) confirming lactoferrin loading of the nanocomplexes. From *left to right*, standard protein ladder, pure bovine lactoferrin (Lf), ZF nanoparticles, Hsp-70 Lf-ZF, Hsp-70 Lf-PEG-ZF and Hsp-70 Ch-Lf-ZF nanocomplexes. Nanocomplex lactoferrin loading was confirmed with specific band detection at 75 kDa

The first phase of this study was the synthesis, characterization and optimization of zinc-doped ferrite nanoparticles (ZF) with their physicochemical properties as the primary criteria in order to procure a highly effective MRI contrast. Selection of zinc, a micronutrient, as the dopant was implemented as a strategy to maximize the characteristic magnetic property of the nanoparticles and simultaneously minimizing potential cytotoxicity in vivo at higher concentrations. Zinc doping reduces the chances of iron overloading by crystallographic ferrite iron cation replacement with highly enhanced saturation magnetization of 108.4 emu/g at room temperature (data not shown), considerably higher than conventional ferrite nanoparticles employed for MRI contrast agent development. Transmission electron microscopy (TEM) revealed the formation of ZF nanoparticles with an average diameter of 24 ± 2 nm (Fig. 1a) and X-ray diffractometry (XRD) confirmed the formation of crystallographic spinel structure (Fig. 1b). The next phase of the study involved the design and development of atherosclerosis-specific nanocomplex MRI contrast agents with emphasis on their in vitro validation by cellular proliferation, toxicity, internalization and molecular targeting assessment. To achieve this goal, three spherical nanosized complexes based on different combinations of ZF nanoparticles, bovine lactoferrin, chitosan and PEG were developed. All nanocomplexes were equipped with heat shock protein (Hsp)-70 antibodies for effective targeting of the atherosclerotic inflammatory site. The three types of engineered nanocomplexes developed were named according to their constituents: Hsp-70 targeted lactoferrin embedded ZF nanoparticles (Hsp-70 Lf-ZF), Hsp-70 targeted chitosan encapsulated lactoferrin embedded zinc ferrite nanoparticles (Hsp-70 Ch-Lf-ZF) and Hsp-70 targeted polyethylene glycol assisted lactoferrin embedded ZF nanoparticles (Hsp-70 Lf-PEG-ZF). Lactoferrin is also a known iron modulator internalized by various cells such as mucosal epithelial cells, hepatocytes, monocytes, macrophages, polymorphonuclear leukocytes, lymphocytes, thrombocytes and fibroblasts due to the presence of a number of cellular receptors including transferrin receptors, Lactoferrin receptors and Lipoprotein receptor-related proteins [18, 23–25]. We used chitosan as, chitosan is a naturally occurring mucoadhesive polysaccharide having a strong affinity for Mucin-1 receptor (Muc-1) expressing endothelial and epithelial cells [26]. Additionally, chitosan as a nanocomplex carrier equips the nanocomplex to undertake transcellular and paracellular internalization pathways and also exhibits strong affinity to cholesterol and triglycerides [27], and lipid accumulation is a primary characteristic of the atherosclerotic plaque inflammation [28]. Moreover, it has also been reported to accelerate the wound healing and exert anti-inflammatory effects [29]. Previously published studies have also shown that dietary chitosan inhibits hypercholesterolaemia and atherogenesis in the apolipoprotein E (apoE) deficient mouse model of atherosclerosis [30]. Orally administered positively charged chitosan binds to lipids in the intestine, blocking absorption, shown to lower blood cholesterol in animals and humans as a result it has been proposed that dietary supplementation with chitosan may inhibit the formation of atherosclerotic plaque [30]. Tail vein injection [31] and intranasal delivery [32] of chitosan based nanoformulations have shown to successfully inhibit atherosclerosis in animal models. However, we were unable to synthesize optimum nanoparticles due to the large polydispersity index (PDI). PEG on the other hand, also has several benefits as it leads to formation of sterically stabilised ‘stealth’ particles [33] and its hydrophilic nature leads to reduced non-specific protein adsorption [34], avoids aggregation [35] and imparts the property of water solubility to the nanoparticles [36]. Therefore, we went on to replace chitosan with PEG and were able to synthesize optimum (224.8 nm, PDI 0.398) nanoparticles. A study performed to assess the distribution of Hsp-70 between fibrotic and necrotic plaques in immunostained carotid endarterectomy specimens revealed that necrotic plaques and their underlying media contained significantly more Hsp-70 expression than the fibrotic tissues [38]. Their results suggested that inadequate Hsp-70 accumulates with smooth muscle cells residing near necrosis to defend against plaque toxicity. Therefore, all nanocomplexes were conjugated with Hsp-70 for target specific internalisation. Hsp-70 is also known to protect cellular components from injury by decreasing oxidation, inflammation and apoptosis and also by refolding damaged proteins [37].

Transmission electron microscopy (TEM) imaging confirmed the formation of all three spherical nanocomplexes (Fig. 1c, e, g). Lactoferrin ZF complex can be clearly observed by the TEM images. Furthermore, lactoferrin ZF complex embedded with chitosan can also be clearly observed by the TEM image. Intensity based hydrodynamic size distribution obtained by dynamic light scattering (DLS) exhibited an average diameter of 224.8 and 275 nm for Hsp-70 Lf-PEG-ZF and Hsp-70 Ch-Lf-ZF nanocomplexes respectively (Fig. 1e, g). A bimodal hydrodynamic size distribution was observed for Hsp-70 Lf-ZF with the primary peak at 435.4 nm and secondary peak at 55.6 nm average diameter respectively (Fig. 1f). Hsp-70 Lf-ZF and Hsp-70 Ch-Lf-ZF nanocomplex exhibited a very high polydispersity index (PDI) value of 0.876 and 0.865 respectively. Hsp-70 Lf-PEG-ZF exhibited a relatively narrow size distribution with a PDI value of 0.398. Furthermore, lactoferrin loading was confirmed by band formation around 78 kDa for all nanocomplexes corresponding to lactoferrin as observed by poly acryl amide gel electrophoresis (PAGE) (Fig. 1i). The serum stability studies performed using DLS confirmed that Hsp-70 Lf-PEG-ZF showed least aggregation in 10 % bovine serum albumin (BSA), followed by Hsp-70 Lf-ZF and Hsp-70 Ch-Lf-ZF nanocomplexs (Additional file 1: Figure S1).

### Measurement of MRI/CT contrast and in vitro internalisation assessment of Hsp-70 Lf-PEG-ZF nanoparticles

**Fig. 2:**
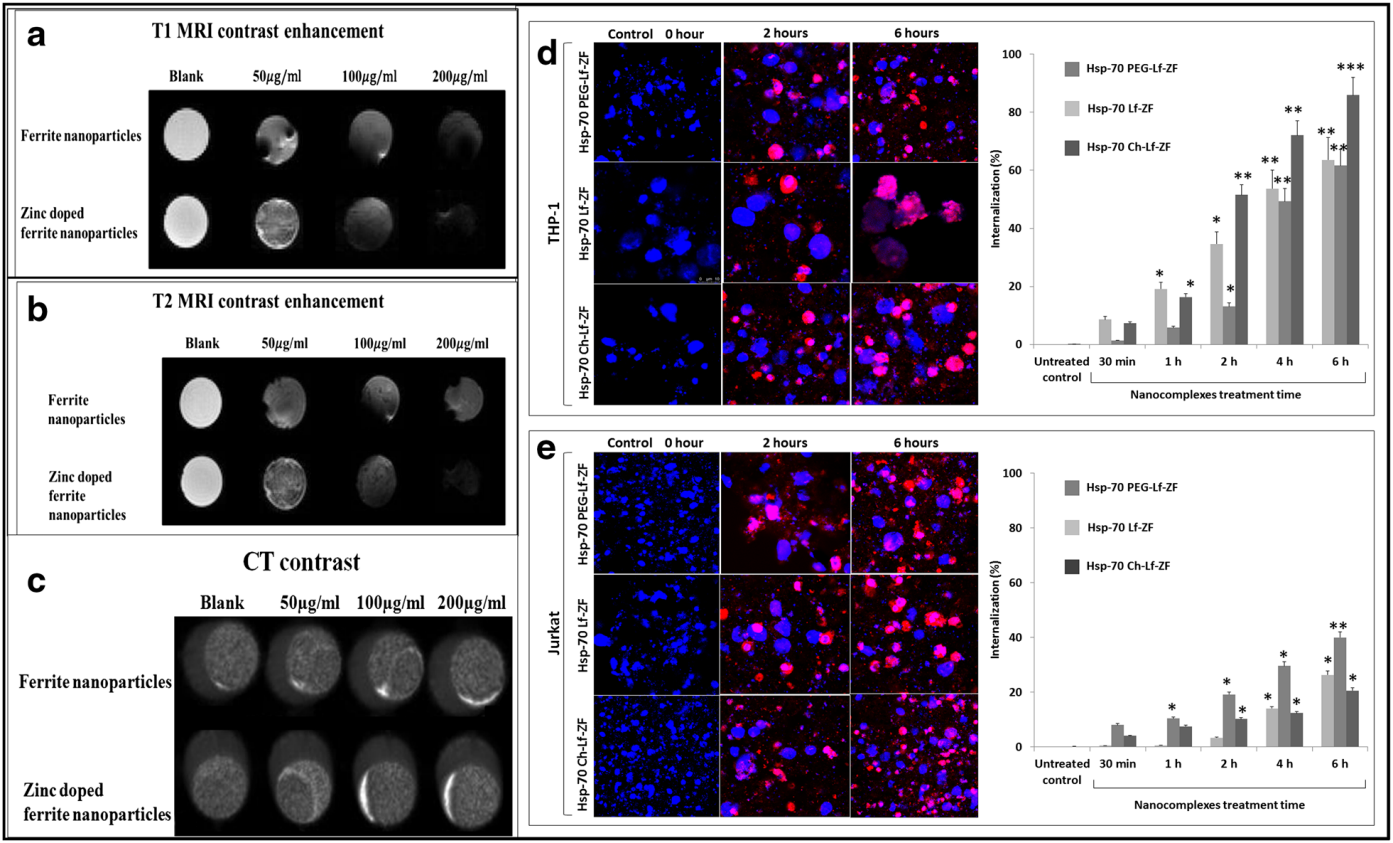
Enhancement in MRI/CT contrast and internalisation efficacy of nanoparticles. **a** Slight enhancement of T1 MRI contrast was observed in Hsp-70 Lf-PEG-ZF compared to ferrite nanoparticles. **b** Significant enhancement of T2 MRI contrast was observed in Hsp-70 Lf-PEG-ZF compared to ferrite nanoparticles. **c** Slight enhancement of CT contrast was observed in Hsp-70 Lf-PEG-ZF compared to ferrite nanoparticles. **d** Confocal microscopy internalization images and cell cytometric internalization assessment displaying time-dependent internalization of Hsp-70 Lf-ZF, Hsp-70 Ch-Lf-ZF and Hsp-70 Lf-PEG-ZF nanocomplexes in THP-1 cells and **e** Jurkat cells. Effective internalization was observed at 2 h Hsp-70 Lf-ZF nanocomplex post-treatment

A slight enhancement of T1 MRI contrast (1.09-fold) was observed in Hsp-70 Lf-PEG-ZF compared to ferrite nanoparticles (Fig. 2a; Additional file 2: Figure S2a). Significant enhancement of T2 MRI contrast (p < 0.05, 1.22-fold) was observed in Hsp-70 Lf-PEG-ZF compared to ferrite nanoparticles (Fig. 2b, Additional file 2: Figure S2b). Slight enhancement of CT contrast (1.08-fold) was observed in Hsp-70 Lf-PEG-ZF compared to ferrite nanoparticles (Fig. 2c, Additional file 2: Figure S2c).

All three nanocomplexes were assessed with THP-1 monocyte and T lymphocyte cell lines. The internalization efficacy of these nanoparticles was tested using fluorescence confocal microscopy and flow cytometry (Fig. 2d, e). Confocal microscopy revealed very effective cellular internalization by all three nanocomplexes at 6 h after treatment with both cell lines. Flow cytometry observation revealed that Hsp-70 Ch-Lf-ZF showed the maximum internalization in THP-1 cells (p < 0.001, 1.41-fold higher than Hsp-70 PEG-Lf-ZF) whereas, Hsp-70 PEG-Lf-ZF showed the maximum internalization in Jurkat cells (p < 0.01, 2.05-fold higher than Hsp-70 Ch-Lf-ZF). This is in accordance with previously published studies where mucoadhesive chitosan nanoparticles have been reported to internalize with very high efficiency in THP-1 cells without inducing any release of pro-inflammatory cytokines [39].

Monocytes and lymphocytes play central roles in atherosclerotic inflammation from the early inflammatory phase to the late thrombotic phase [40]. Therefore, in this study, the nanocomplex effects and internalisation were studied with THP-1 monocytes and Jurkat lymphocytes as in vitro models of cells having a central role in atherosclerotic plaque inflammation [28].

Flow cytometry also revealed highly efficient internalization by monocytes as compared to lymphocytes primarily due to their Phagocytic characteristic. LDH release (cytotoxicity) and CyQUANT cell proliferation assays were performed in both THP-1 and Jurkat cells following treatment with all three nanocomplexes (Additional file 3: Figure S3a–c). The results revealed that Hsp-70 PEG-Lf-ZF failed to induce any significant cytotoxicity but led to a significant cellular proliferation (p < 0.05) with both THP-1 or Jurkat cells in lower concentrations (40–2.5 µg/ml). The Hsp-70 Ch-Lf-ZF led to significant cytotoxicity and significant reduction in proliferation (p < 0.05) in both THP-1 and Jurkat at 100 µg/ml and led to significant (p < 0.05) increase in proliferation in THP-1 cells at lower concentrations (50–2.5 µg/ml).

### Ex vivo MRI/CT of Hsp-70 Lf-PEG-ZF nanoparticles in *P. obesus* atherosclerotic model

*Psammomys obesus* exhibit age-dependency and phenotypic heterogeneity in acquiring diabetes and obesity indicating the polygenic nature of pathogenesis in *P. obesus*, closely mimicking the human populations [41]. A genetic correlation between type-2 diabetes, atherosclerosis and inflammation has been well established since the discovery of the role of Tanis gene in *P. obesus* [22]. Since, we found that Hsp70-Lf-ZF was not suitable due to large particle size and Then we syntheized Hsp-70 Ch-Lf-Zf nanocomplex had very high poydispersity index. Therefore, all further studies were conducted with Hsp70-PEG-Lf-ZF that has a narrower hydrodynamic size distribution.

Therefore, the next phase of ex vivo imaging experiments emulated a real-time in vivo scenario by injecting Hsp-70 Lf-PEG-ZF contrast agent directly into the intact animal aorta. Three groups of rats (18 months old Control n = 5, 6 months old injected with Hsp-70 Lf-PEG-ZF n = 5 and 16 months old injected with Hsp-70 Lf-PEG-ZF n = 5) were used with minimal dissection steps prior to MRI/CT scan. Several benefits can be realized by using a direct intra-aortic ex vivo contrast agent injection strategy. It imitates an in vivo MRI scan of the animal providing additional internal anatomical information from MRI contrast interferences arising from organs, tissues and other materials and fluids, especially fat. Furthermore, the strategy minimizes the risk of causing any damage to the aorta and heart prior to imaging. There is minimal dissection, and pre-treatment electrocautery steps to seal aortic branches can be avoided with no possibility of any unwanted artefacts. The exploratory ex vivo MRI/CT assessment reported in this study paves the way for future in vivo multi-modal imaging studies with *P. obesus* for development of atherosclerotic inflammation specific contrast or therapeutic agents.

**Fig. 3:**
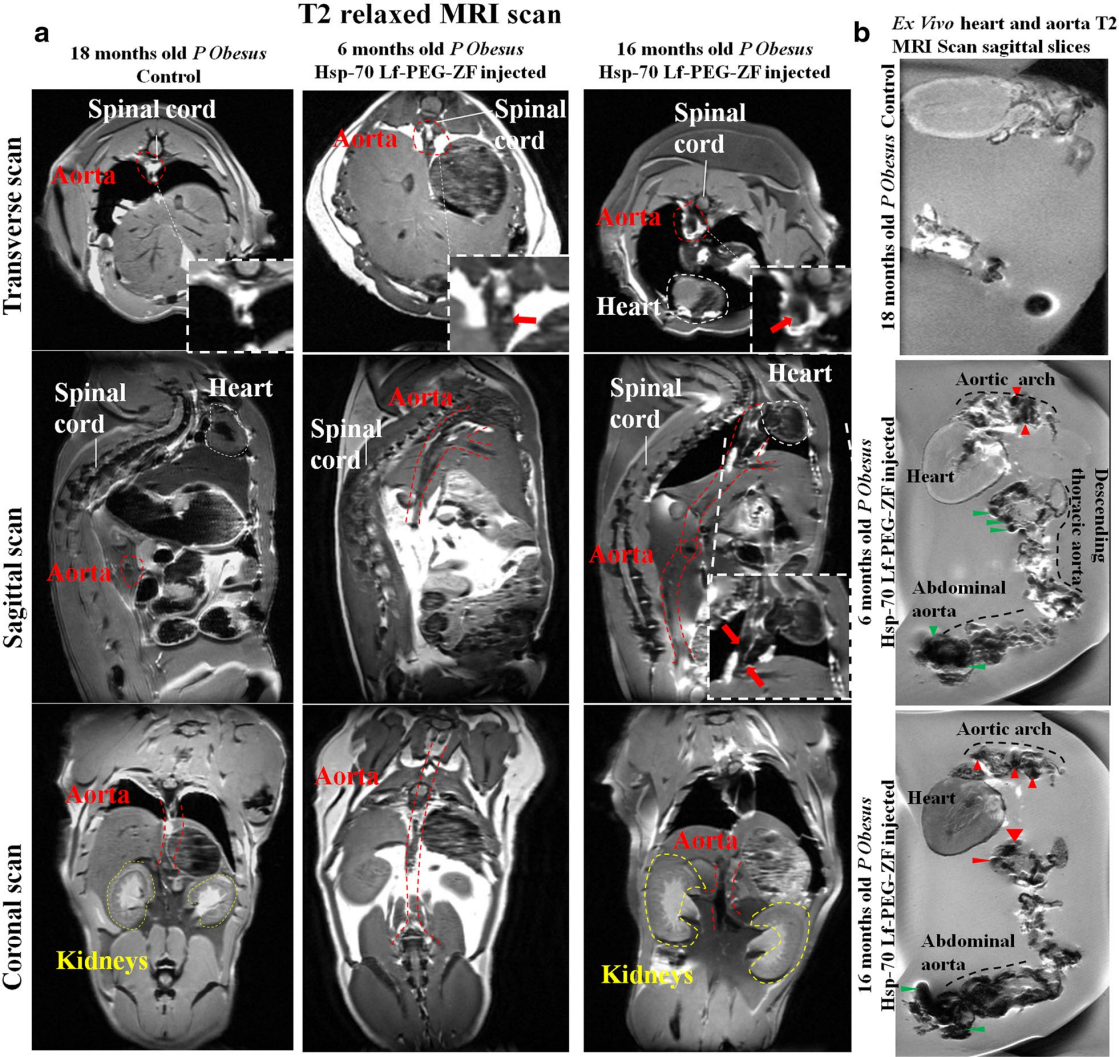
T2 relaxation MRI scan imaging of Hsp-70 Lf-PEG-ZF nanocomplex contrast agent injected *P. obesus* aortae and hearts. **a** Six (n = 5) and 16 months old (n = 5) rats were injected with Hsp-70 Lf-PEG-ZF nanocomplex contrast agent. Eighteen month old rats (n = 5) were maintained as control. Representative images were presented in transverse, sagittal and coronal orientation respectively. *Red*, *yellow* and *white* *dotted lines* outline the aorta, kidney and heart respectively. The spinal cord has also been pointed in white. The *red arrows* represent the atherosclerotic plaque site at the aortic arch region. **b** Shortlisted and magnified fat saturated T2 relaxation sagittal images showed high intensity site-specific MRI contrast that was attributed primarily due to disrupted aortic intimal structure possibly due to atherosclerotic pathological conditions like endothelial denudation, macrophages and foam cell entrapment, necrosis and calcification. High MRI contrast regions are indicated with *red arrows*

Representative images (Fig. 3a) and the corresponding heat map images (Additional file 4: Figure S4a) were presented for simplification. The control animal, perfused with PBS only showed minimal MRI intensity in all three orientations (Transverse, sagittal and coronal). High positive contrast exhibiting peri-aortic fat accumulation was clearly observable around the arch region. The renal artery had a poorly defined periphery with the sagittal scan images. Transverse scan images provided the most prominent information about the aortic peripheral morphology with the contrast agent injected in young and old animals. However, with the PBS injected animal the periphery was very poorly defined to derive any conclusion about *P. obesus* cardiovascular health status. A detailed examination of the aortic morphological status was conducted with the MRI assessment with both young (6 months old) and old (16 months old) direct Hsp-70 Lf-PEG-ZF agent injected *P. obesus*. A higher overall aortic contrast intensity (p < 0.05, 1.71-fold) was observed in the old animals compared to the younger counterparts (Additional file 4: Figure S4b). However, both animals displayed atherosclerotic narrowing with intense T2 relaxed negative contrast at the descending aortic arch site adjacent to the high positive contrast peri-aortic fat accumulation. This mixed positive negative contrast at the arterial narrowing sites is caused by negative MRI contrast signal arising from accumulated contrast agent and positive contrast exhibited by lipid at the atherosclerotic plaque site. The young animal MRI images exhibited moderate site-specific accumulation only at the aortic arch site with arterial stenosis and the remaining tissue. Alternatively, the older animal displayed strong contrast enhancement at the aortic arch and descending thoracic aorta regions and moderate contrast at the abdominal aorta.

MRI scan of the aorta from these rats exhibited moderate contrast enhancement at the aortic arch and significantly high contrast enhancement at the descending thoracic aorta region compared to the control. The nanocomplex contrast agent injected heart and aorta agar phantom was made in a customized diamagnetic holder to avoid any scan interference and imaging was performed with a 9.4T MRI scanner (Fig. 3b; Additional file 5: Figure S5). Overall, excellent T2 MRI contrast enhancement was observed with the contrast agent injected old animal aortas compared to the young animal aortas and the control.

**Fig. 4:**
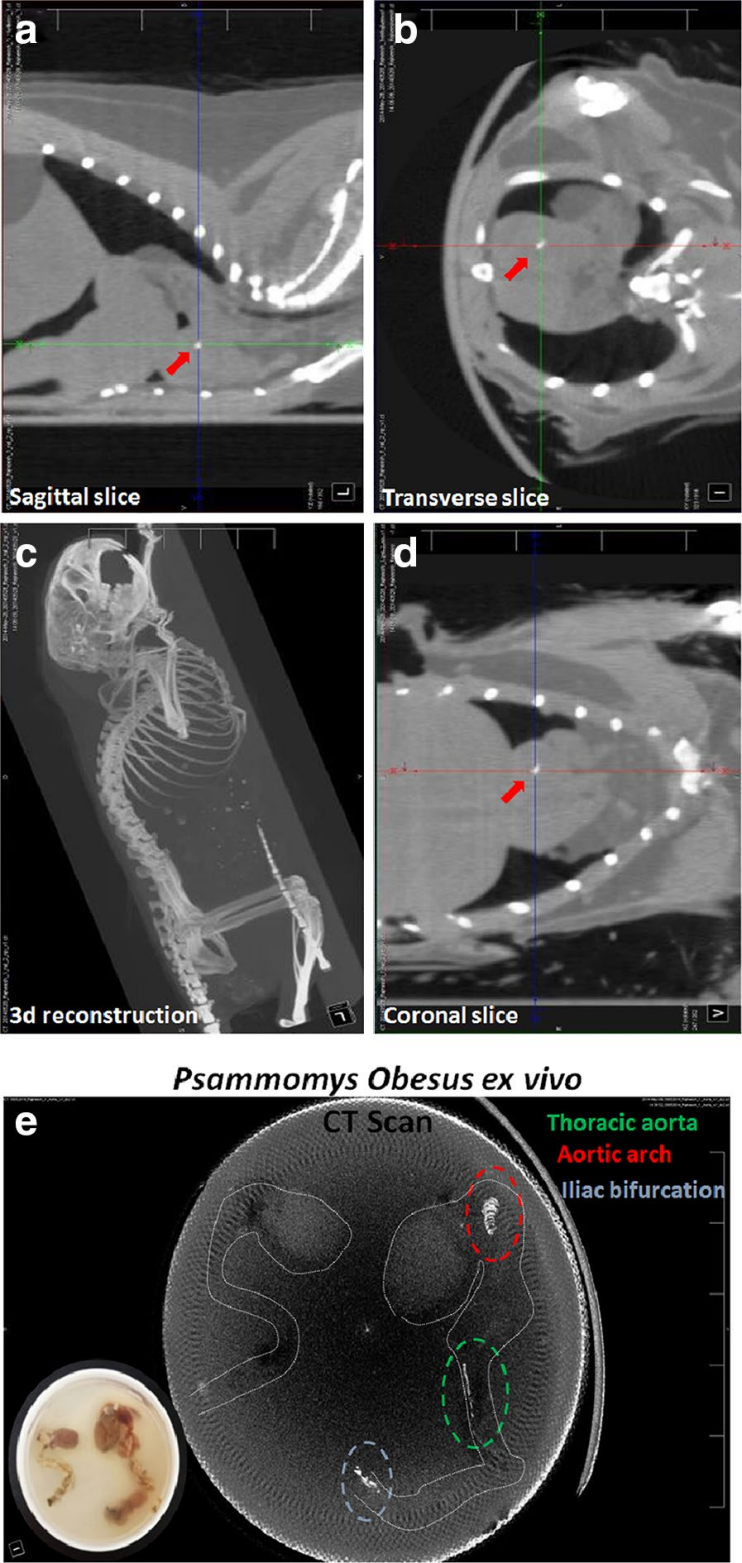
X-ray computed tomography scan images of intra-aortic Hsp-70 Lf-PEG-ZF contrast agent injected 16 month old *P. obesus*. **a** Sagittal, **b** coronal, **c** transverse and **d** 3d reconstructed CT images. *Red arrows* indicate the streak at the aortic arch site with Hsp-70 Lf-PEG-ZF nanocomplex accumulation. **e** Three dimensional reconstruction image generated with the Hsp-70 Lf-PEG-ZF nanocomplex injected *P. obesus* hearts and aortae agar phantom CT scan (*inset*). The periphery of the cardiovascular tissue was outlined with a *dotted white line* following a tissue outline observed with the CT image. Aortic regions with enhanced CT contrast are evident in the aorta and highlighted by *red and blue dotted lines* representing the aortic arch and terminal iliac bifurcation respectively

Additionally, contrast agent injected 16 months old (adult) *P. obesus* were subjected to X-ray CT scan to assess aortic arch contrast agent localization corresponding to the T2 MRI scan. Sagittal, transverse and coronal scan exhibited a clear streak with intense CT signal the aortic arch site due to targeted accumulation (seen in white contrast) at the aortic arch site reaffirming the nanocomplex contrast agent accumulation at the aortic arch site (Fig. 4a–d). This was followed by CT imaging of the aorta to assess the potential for CT bimodality. The analysed aortic regions with T2 MRI contrast enhancement image directly corresponded with the three dimensional (3D) CT scan. The CT contrast enhancement at the aortic arch, thoracic aorta and iliac bifurcation region of the 16 month old animal precisely correlated with the MRI scan assessment, whereas the juvenile 6 month old animal did not exhibit any significant CT contrast enhancement intensity relating to moderately MRI contrast enhancement (Fig. 4e).

### Site specific internalisation and biomarker analysis using histological studies

Histological analysis of aorta and heart tissues of Hsp-70 Lf-PEG-ZF nanocomplex injected rats was performed and compared with control rats to visualize the contrast agent distribution and assess the severity of atherosclerotic inflammation.

**Fig. 5:**
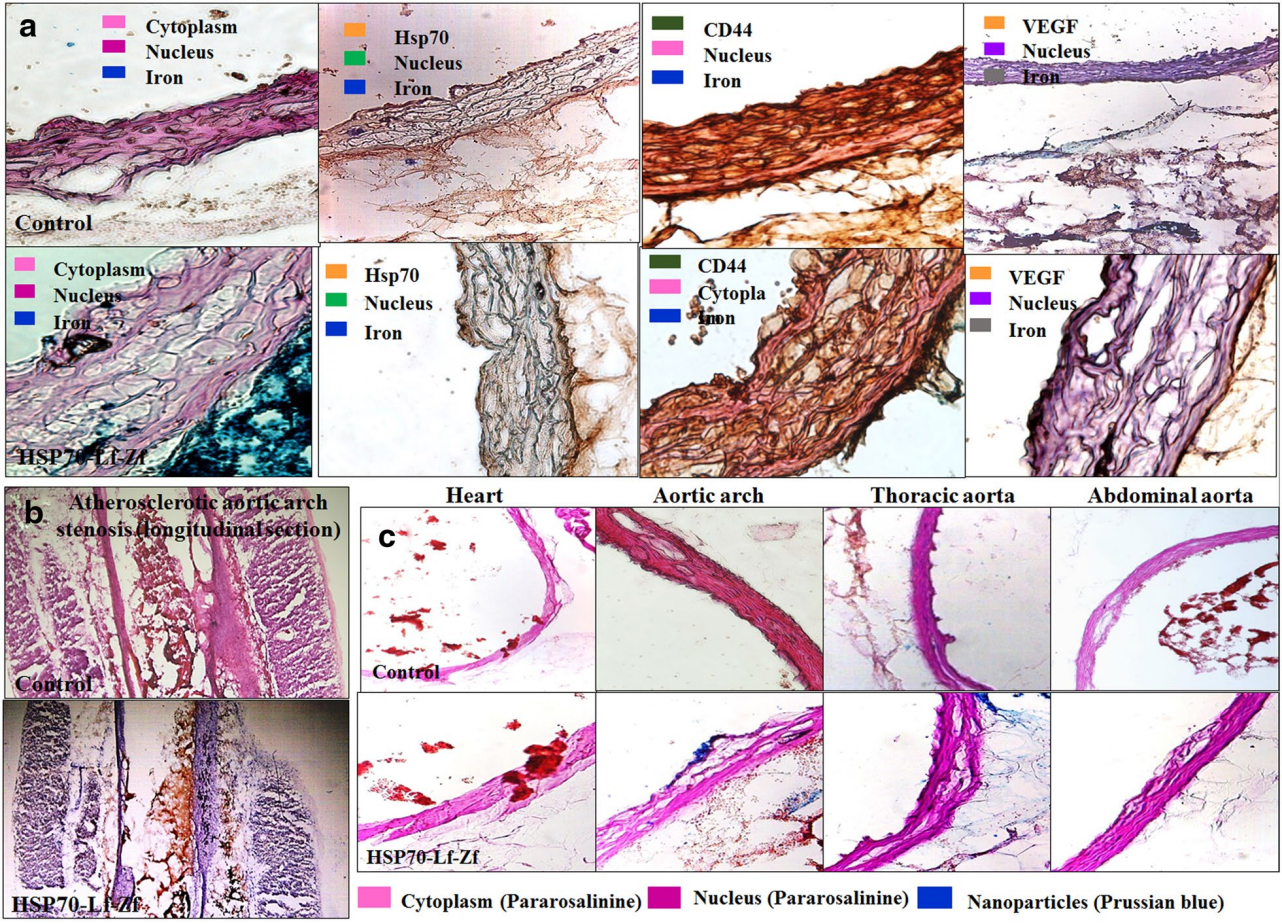
Histological assessment images for nanoparticle distribution and vascular morphology assessment of intra-aortic Hsp-70 Lf-PEG-ZF nanocomplex contrast agent injected *P. obesus* aorta and heart sections. Nanoparticle distribution images of axially sectioned captured at ×20 magnification and the tissues were and stained with Prussian blue iron stain and Pararosalinine cellular and nuclear stain. **a** Immunohistological images of axially sectioned aortic arch from control rats and Hsp-70 Lf-PEG-ZF injected rats, stained against Prussian blue iron stain, Hsp-70, CD44 and VEGF respectively captured at ×20 magnification. **b** Longitudinal section of aortic arch tissue arch from control rats and Hsp-70 Lf-PEG-ZF injected rats stained against hematoxylin nuclear and oil red-O lipid stain captured at ×10 magnification. Advanced atherosclerotic plaque causing severe luminal stenosis was observed at the aortic arch region. Pathological intimal thickening with significant lipid deposition within the intima, media, necrotic core as well as extracellular lipid accumulation in the aortic lumen was observed. **c** Nanoparticle accumulation could not be observed in the heart with no disruption of the pericardium. Aortic arch section displayed severely disrupted intimal structural integrity and widening of first interlamellar spaces with atherosclerotic micro lesions. The nanoparticles can be seen attached with the intimal endothelium, within the intima and across into the adventitia. Descending thoracic aorta with complex micro lesions displayed significant nanoparticle accumulation within the intima and adventitia. Intima widening could be observed with *blue steaks* in the intima indicating cellular internalization and interlamellar contrast agent accumulation. The iliac bifurcation section adjacent to the abdominal aorta with partly disrupted intimal integrity and fewer lesions was observed. The endothelial layer was mostly conserved with minimal endothelium denudation. Adventitial nanoparticulate contrast agent accumulation was also minimal abdominal aortic region. Number of micro-atherosclerotic lesions and intimal widening was significantly less in comparison to aortic arch and descending thoracic aorta. Hematoxylin, Methyl green and Pararosalinine were used for nuclear staining and antibodies were stained with 3,3'-Diaminobenzidine DAB

Multiple cell types at the atherosclerotic aortic arch region including endothelial cells, smooth muscle cells and macrophages exhibited Hsp-70 overexpression (Fig. 5a). Aortic endothelium primarily expresses Hsp-70 along with other intimal cells at atherosclerotic inflammatory sites in the atherosclerosis rat model (ApoE^−/−^), and a primate model (macaque) as well as humans (post-mortem) [20, 42]. Complex atherosclerotic lesions were observed with foam cells and debris surrounded by extracellular matrix and smooth muscle cells in the interlamellar space. Stretch injured intima, endothelial denudation and loss of internal elastic lamina integrity was prevalent at the aortic arch region. Prevalent stretch induced intimal injury, endothelial denudation and loss of internal elastic lamina was observed throughout the aortic arch region. Cellular contrast agent internalization was observed with intimal migratory and non-migratory smooth muscle cells. Intimal macrophage accumulation at the aortic arch site also displayed significant contrast agent accumulation. Widespread endothelial denudation and disrupted elastic lamina was observed in at the aortic arch region. Disrupted endothelium, migrating smooth muscle cells and macrophages extensively expressed stress protein, mainly at the lumen and adventitial side of the intima. Smooth muscle cell migration and cell debris and apoptotic cells were observed all along the intimal region. Angiogenesis growth factor was mostly expressed by the intimal endothelium layer at the luminal side and migratory smooth muscle cells at the adventitial side of the intima. Longitudinally sectioned aortic arch displayed pathologically mature translational plaque protruding in the lumen with severe intimal thickening, lipid deposition, necrosis, fibrous cap formation and calcification.

The contrast agent injected adult animals exhibited severe intimal disruption and widening accompanied with collagen rich plaques with fatty streaks or intimal foam cell accumulation without necrosis along the entire length of the aorta. Luminal stenosis was accompanied by pathological intimal thickening with collagen rich xanthomas with early and late necrotic sites observed throughout the aorta, conditions predominantly responsible for cerebral and myocardial infarction (Fig. 5b). Extensive lipid deposition was realized within the luminal plaque, intima and the necrotic core. Contrast agent exhibited selective accumulation at the occluded aortic arch region within the intima, necrotic core and adventitia at the atherosclerotic inflammatory site validating the target-specific delivery of the developed Hsp-70 Lf-PEG-ZF nanocomplex MRI contrast agent (Additional file 6: Figure S6).

**Fig. 6:**
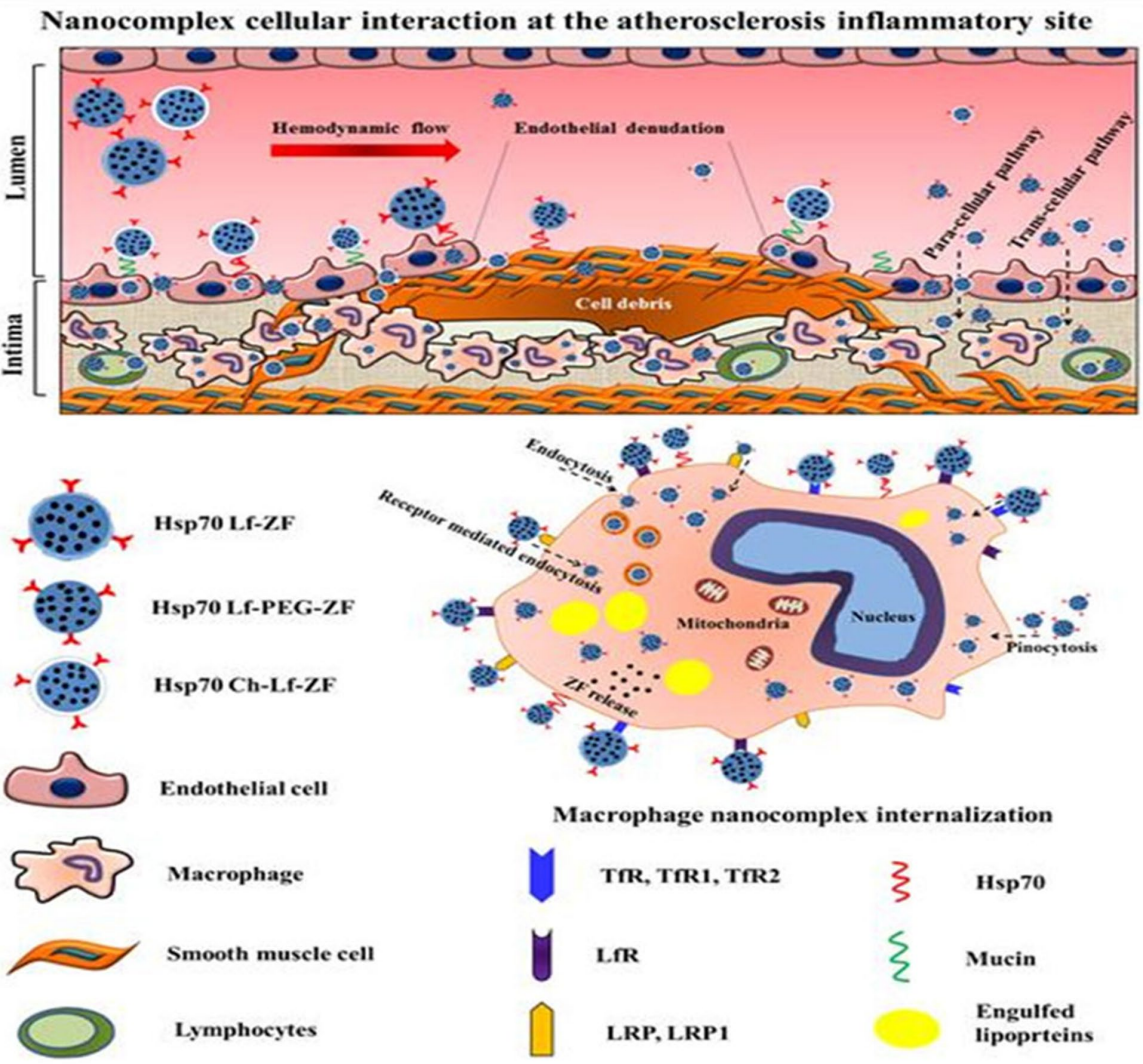
Schematic representation of different mechanisms of passive and targeted nanocomplex delivery and cellular internalization at the atherosclerotic inflammatory region. The diagram schematically represents targeted and passive delivery and cellular interaction of nanocomplexes (Hsp-70 Lf-ZF, Hsp-70 Lf-PEG-ZF and Hsp-70 Ch-Lf-ZF) at the site of atherosclerotic inflammation. The nanocomplexes in the luminal hydrodynamic flow have been shown to enter the intimal layer due at the plaque site due to endothelial denudation or leaky vasculature. The Ch-Lf-ZF nanocomplex has been represented to cross the intimal endothelial layer by paracellular and transcellular pathways and also interacting with the mucin receptors followed by internalization. The cells under stress at the plaque sites exhibit cytoprotective Hsp-70 overexpression and therefore the Hsp-70 targeted nanocomplexes have been shown to interact with multiple cell types followed by internalization. Lactoferrin mediated cellular interaction and receptor-mediated (TfR, TfR1, TfR2, LfR, LRP, LRP1) internalization has been shown with a representative macrophage cell (enlarged). Also, Lactoferrin potentially interacts with the macrophage scavenger receptor saturating further lipoprotein uptake by the macrophages and preventing further inflammation. The nanoparticles also exhibiting endocytosis and pinocytosis has also been represented. The nanocomplexes finally breakdown and the zinc ferrite nanoparticles released within the cells

The heart sections demonstrated highly conserved pericardium structure with contrast agent internalization and no accumulation into the myocardium (Fig. 5c). Myocardial pericardium and aortic endothelium exhibited nanoparticle internalization throughout the cardiovascular tissue, exhibiting an overall negative T2 MRI contrast generating images of the entire structure. The aortic arch and descending thoracic aorta exhibited severe contrast agent accumulation across the intima and media into the adventitia, attributed primarily to diffusion through atherosclerotic disrupted aortic intimal and medial structure. It should be noted that altered endothelium permeability, function and expression is accelerated by irregular hemodynamic flow at the sites of arterial bifurcation and curvature [43–45]. The contrast agent can therefore be targeted to the atherosclerotic inflammatory site by passive diffusion through the damaged intima as well as active targeting by lactoferrin and Hsp-70 multi-cellular receptors (Fig. 6). Collagen-rich plaques with significant luminal stenosis were prevalent throughout the aorta. Healed erosions or ruptures led to large areas of calcification with inflammatory cells and minimal or absence of necrosis. Stretch induced intimal injury, endothelial denudation, loss of elastic lamina, smooth muscle cell migration, macrophage and apoptotic cell accumulation were clearly visible at the complex aortic arch micro-angiogenesis region.

## Conclusion

The study demonstrated development of engineered atherosclerosis targeted ZF nanoparticle nanocomplex based T2 relaxation MRI contrast agent which was validated in an ex vivo setting. We also observed that lactoferrin receptor and Hsp-70 antibody induced atherosclerotic inflammatory site specific multi-cellular targeting. Additional insight about aortic contrast agent distribution, accumulation, targeting and internalization was gained by histological examination with both longitudinally and axially oriented aorta and heart sections of contrast agent injected *P. obesus* rat. Particularly, likely disruptions in the aortic intimal structure due to hemodynamic stress around bifurcation sites were identified, especially in the aorta, a characteristic feature of atherogenesis leading to leaky vasculature. The direct intra-aortic contrast agent injection strategy effectively avoids any artefacts and negates the risk of any damage to the aorta. Juvenile *P. obesus* (6 months old) aorta and heart images present a significantly lower overall T2 MRI contrast enhancement in comparison to the adult animals (16 months old), attributed to more conserved and intact intimal endothelium structure. The adult *P. obesus* aorta and heart MRI images displayed high overall T2 negative contrast. Significant site-specific T2 MRI contrast was observed at the aortic arch, abdominal thoracic aorta and iliac bifurcation region indicating increasingly disrupted aortic peripheral structure and multi-molecular cellular targeting at these sites. The CT image verified site-specific contrast enhancement observed by aortic T2 MRI scans of 16 month old *P. obesus* where a significant contrast was observed at the aortic arch, thoracic aorta and iliac bifurcation. The point to be noted here is that not the entire contrast enhancement observed by MRI was reflected in the CT scan. Therefore, the CT contrast is generated only at the specific sites with significant contrast agent accumulation, requiring a high concentration to produce effective CT contrast. Accumulation of MRI contrast agent at atherosclerosis inflammation prone sites, specifically the aortic arch, descending thoracic aorta and iliac bifurcation in adult *P. obesus*, was reaffirmed by the 3D reconstructed CT image. MRI contrast generation is an intricate process and requires manipulation of several parameters in order to produce contrast depending on the proton spin of the material under external magnetic fields. On the other hand, X-ray CT is quite straight forward and is based on electron density of the material. On striking the material the X-rays pass through low electron density material with no loss of intensity as observed by a receiving detector. When the electron density is sufficiently high due to contrast agent accumulation, the X-ray shows a change in intensity at the receiver registered as contrast. In the case of Hsp-70 Lf-PEG-ZF injected cardiovascular tissue of 16 month old rats, the CT contrast enhancement was noted at various sites due to contrast agent accumulation. As mentioned earlier, the contrast agent accumulation at these sites could be due to several factors, primarily disruption in the aortic intima leading to a leaky vasculature, targeted and passive nanoparticle internalization. The factors leading to accumulation at specific aortic sites was successfully evaluated by thorough histological analysis. Site-specific T2 relaxation signal intensity exhibiting contrast agent would require considerably reduced administrative dose for highly effective diagnosis of atherosclerosis. This is the first ever study reporting enhanced ex vivo bimodal CT contrast with zinc-doped ferrite nanoparticle nanoparticles based MRI contrast agent identifying unexplored new avenues in cardiovascular imaging research. *Ex vivo* CT/MRI contrast enhancement using Hsp-70 Lf-PEG-ZF contrast agent showed promising results opening multimodal applicability avenues for ferrite nanoparticle based contrast enhancement.

The imaging experiments reported in this study imitated a real-time in vivo scenario by injecting Hsp-70 Lf-PEG-ZF contrast agent directly into the intact animal aortas with minimal dissection steps prior to MRI/CT scan. Several benefits can be realized by using direct intra-aortic ex vivo contrast agent injection strategy. It imitates an in vivo MRI scan of the animals providing additional internal anatomical information from MRI contrast interferences arising from organs, tissues and other materials and fluids, especially fat. The contrast agent validation and atherosclerosis-specific targeting in this study justifies further studies in order to strategically observe the contrast agent in in vivo scenarios. Furthermore, the strategy minimizes the risk of causing any damage to the aorta and heart prior to imaging. The exploratory ex vivo MRI/CT assessment reported here paves way for future in vivo multi-modal imaging studies with *P. obesus* for development of atherosclerotic inflammation specific contrast agents with potential for therapy.

## Methods

### Synthesis of zinc ferrite (ZF) nanoparticles

An optimized batch of ZF nanoparticles Zn_0.4_Fe_2.6_O_4_ was synthesized using a hydrothermal chemical synthesis technique. Laboratory reagent grade ferric chloride (FeCl_3_·6H_2_O), ferrous chloride (FeCl_2_·4H_2_O), zinc chloride (ZnCl_2_) and analytical reagent grade sodium hydroxide (NaOH) were purchased from Ajax Fine Chemicals (Australia). FeCl_3_·6H_2_O 0.125 M was dissolved with calculated amounts of 0.125 M FeCl_2_·4H_2_O and 0.125 M ZnCl_2_ in de-oxygenated and de-ionised water to obtain an aqueous solution with Fe^3+,^ Fe^2+^ and Zn^2+^ ions respectively. The solution was stirred at 200 rpm and maintained at room temperature followed by drop wise addition of 1 M NaOH reducing agent. The suspension was immediately autoclaved for annealing at 150 °C for 12 h. The precipitated zinc doped ferrite nanoparticles were washed multiple times by magnetic decantation. Subsequently, the nanoparticles were freeze dried at −80 °C and 0.013 mbar pressure for 24 h.

### Synthesis of nanocomplexes

For the synthesis of Hsp-70 Lf-ZF nanocomplex, 20 mg of optimized ZF nanoparticles were dispersed in 10 ml deionised water and ultrasonicated at high intensity for 30 min. Dopamine, 2 mg/ml (Sigma Aldrich) was added to the nanoparticle aqueous suspension and ultrasonication was continued for another 30 min. The particles were washed by magnetic decantation and redispersed in 10 ml water and stirred at 200 rpm at 4 °C. 2.7 ml 1-Ethyl-3-(3-di-methylaminopropyl) carbodiimide (EDC) (2 mg/ml) (Sigma Aldrich) was added to the nanoparticle suspension followed by addition of 3.4 ml N-hydroxysulfosuccinimide (NHS) (Sigma Aldrich) (2 mg/ml) at 45 min and the aqueous suspension was allowed to stir overnight at 4 °C (EDC/NHS coupling). The unreacted coupling agents were removed by magnetic decantation followed by addition of 5 mg/ml bovine lactoferrin solution to the activated nanoparticles with continuous stirring for 12 h. The nanocomplex was collected by centrifugation at 5000 rpm for 30 min. Lactoferrin zinc ferrite (Lf-ZF) nanocomplexes were redispersed in milliQ water and reactivated by EDC/NHS coupling reaction (Additional file 7: Figure S7). The reaction was quenched by adding 10 µl β-mercaptoethanol followed by addition of 10 ml heat shock protein antibody (Hsp-70) (Sapphire Biosciences) in water (1:1000) and stirred at 200 rpm at 4 °C for 12 h. The final antibody nanoparticle complex was recovered by centrifugation at 5000 rpm for 30 min and freeze dried at −80 °C and 0.013 mbar pressure. Hsp-70 Lf-ZF nanoparticle sample was obtained in powder form and used for further characterization and experiments. Synthesis of Hsp-70 Lf-PEG-ZF involved dispersion of 200 mg ZF nanoparticles in ultrapure water and ultrasonication for 30 min. This was followed by addition of 200 mg carboxylated polyethylene glycol (PEG) (molecular weight 1000) (JenKem, USA) and ultrasonication was continued for 30 min at 80 % amplitude. The particles were washed by magnetic decantation and redispersed in 10 ml water and stirred at 200 rpm at 4 °C. 2.7 ml EDC (2 mg/ml) (Sigma Aldrich) was added to the nanoparticle suspension followed by 3.4 ml NHS (2 mg/ml) after 45 min and the aqueous suspension was allowed to stir overnight at 4 °C. The unreacted coupling agents were removed by magnetic decantation followed by addition of 10 ml aqueous solution containing 5 mg bovine lactoferrin. After 12 h, the nanocomplex was collected by centrifugation at 5000 rpm for 30 min. The nanoparticles were redispersed in 10 ml water followed by EDC/NHS coupling and 50 µl β-mercaptoethanol followed by addition of 10 ml Hsp-70 antibody in water (1:1000) and stirred at 200 rpm at 4 °C for 12 h (Additional file 8: Figure S8). The nanocomplex was recovered by centrifugation at 5000 rpm for 30 min and the pellet was frozen in liquid nitrogen and freeze dried at −80 °C and 0.013 mbar pressure. The chemical reaction and synthesis steps have been schematically described in supplementary information. For the synthesis of Hsp-70 Ch-Lf-ZF nanocomplex, 40 mg ZF nanoparticles were dispersed in 10 ml ultrapure milliQ water and ultrasonicated at high intensity for 30 min. Ultrasonication was continued for another 30 min in 10 ml of 2 mg/ml aqueous dopamine solution. The nanoparticles were washed by magnetic decantation and redispersed in 10 ml milliQ water. 2.7 ml of 2 mg/ml EDC (Sigma Aldrich) was added to the nanoparticle suspension followed by addition of 3.4 ml 2 mg/ml NHS (Sigma Aldrich) (2 mg/ml) to initiate the coupling reaction stabilizing the amine reactive intermediate at 45 min and the aqueous suspension was allowed to stir overnight at 4 °C. The nanoparticles were recollected by centrifugation at 5000*g* for 30 min and redispersed in 90 ml water with 200 mg chitosan. The solution was stirred and maintained at 4 °C with constant stirring at 200 g. 10 ml of 2 mg/ml Sodium tri-polyphosphate solution in acetate buffer was added to the working solution drop wise and the solution was allowed to stir for 2 h. The solution was centrifuged at 5000*g* for 30 min and the pellet was collected and redispersed in 10 ml water followed by EDC/NHS activation and the coupling reaction followed quenching using 10μl β-mercaptoethanol. 10 ml of 1:1000 Hsp-70 antibody was added drop wise to the solution and stirred overnight. The nanocomplex solution was washed twice and collected by centrifugation at 5000 rpm for 10 min. Finally the nanocomplex was freeze dried at −80 °C and 0.013 mbar pressure. All nanocomplexes were stored at 4 °C temperature.

### Characterization procedure for nanoparticles

TEM assessment of nanoparticle and nanocomplex characterization was performed with JEOL JEM-2100^®^ LaB6 transmission electron microscope. All samples were prepared on carbon coated 400 mesh GSCU400C-50 Formvar^®^ Copper grids and simultaneous sequentially imaging was performed. Standard operating protocol was followed with constant 200 kV high tension voltage and 110 µA beam current. Hydrodynamic size and zeta potential measurements were performed with Malvern Zetasizer Nano^®^ ZS. Crystallographic assessment was performed with Panalytical X’Pert^®^ PRO X-ray diffractometer. Freeze dried nanoparticle powder was placed in the sample holder and the top surface layer was smoothened. The scans were performed from 10° to 70° 2θ X-ray beam angle with 0.02 unit step size per 2 s. For PAGE, equal amounts of protein and lactoferrin nanocomplex (with 5× loading dye) were subjected to Sodium Dodecyl Sulfate-Poly acrylamide gel Electrophoresis (200 V, 3A, 300 W) on 10 % gel to visualize the bands. Polyacrylamide gel contained 1.5 M (pH 8.8) and 1 M (pH 6.8) Tris buffer (Sigma Aldrich) for separating and stacking gel respectively, 30 % Acrylamide (Bio-Rad), 10 % Ammonium Persulfate (APS) (Bio-Rad) and 0.2 % TEMED (Sigma Aldrich). The gel was treated with Coomassie staining solution for 1 h followed by de-staining solution treatment for 30 min. The gel was imaged using Bio-rad Chemidoc XRS and the bands were assessed.

The nanocomplexes were dissolved in 10 % BSA in a concentration of 1 mg/ml until a period of 7 days and accumulation in the respective nanocomplexes was observed using DLS at regular time intervals. The T1, T2 and CT contrast of the Hsp-70 Lf-PEG-ZF nanocomplex was compared with commercially available ferrite nanoparticles. Equal weight of both nanoparticles was set in 1 % agar in a customized diamagnetic sample holder for nominal magnetic resonance response and the contrast was observed. The MRI was performed at the Monash biomedical imaging centre, Melbourne. The Agilent 9.4 T MRI small animal scanner was used for this study. This instrument is equipped with 1H 63/108 mm coil designed as a standard 16-element coil with 6 cm sample diameter, which is suitable for small animal imaging. The T1 and T2 MRI signal intensities of nanoparticles were measured and analysed using the Vnmr J4 software. The CT scans were obtained using the SOMATOM CT scanner (Siemens Australia) also at the Monash Biomedical Imaging Centre, Melbourne.

### In vitro methodologies used

Human THP-1 monocytes (ATCC^®^ TIB-202™) and Jurkat T-lymphocytes Clone E6-1 (ATCC^®^ TIB-152™) cell lines were obtained from American type culture collection (ATCC), Virginia, United States of America (USA). Both cell lines were grown in Roswell Park Memorial Institute (RPMI) 1640 media supplemented with Dulbecco’s Modified Eagle Medium (DMEM) obtained from Gibco™ Life Technologies (Australia) and 1 % penicillin/streptomycin (P/S). The cells were maintained at 37 °C and 5 % CO_2_ in a humidified incubator (Heracell^®^ 150i, Thermo Scientific, Australia). The cells were regularly subcultured in 25 and 75 cm^2^ cell culture flasks purchased from Greiner BioOne™ (Australia) after attaining 80–90 % confluence. Being non-adherent cell lines, the suspension cells were directly collected and centrifuged at 1200 rpm for 5 min. After centrifugation, the cell viability was estimated by trypan blue haemocytometer count using a microscope and 1 × 10^5^ cells/ml were resuspended in flasks with fresh media.

### Cytotoxicity studies using lactate dehydrogenase (LDH) assay

The cytotoxic effects nanocomplexes were assessed by lactate dehydrogenase (LDH) detection assay kit (Roche, Australia) according to the manufacturer’s instructions. Cells were incubated in 96 well plates at a cell density of 1x10^5^cells/ml and incubated for 24–48 h at 37 °C in a humidified 5 % CO_2_ atmosphere. Post-incubation the cells were incubated with all the treatments prepared in 1 % serum supplemented RPMI medium. The cells were then incubated at 37 °C for 24 h. Controls were set up as per the manufacturer’s instructions, with a positive control using 100 µl cells with 10 % serum supplemented media, 100 µl 1 % Triton-X 100 (Sigma Aldrich, Australia) as negative control and treatments containing 100 µl of cells grown in 1 % serum supplemented media. After incubation the plate was centrifuged at 4800 rpm for 5 min at room temperature to settle the cells and debris. The supernatant from each well was then collected and transferred to a fresh 96 well plate. To this supernatant, freshly prepared 100 µl of standard LDH reaction solution was added and the plate was incubated in the dark for 30 min at room temperature. After incubation the absorbance was measured using a micro plate reader (Corona electric) using a filter of 492 nm with a reference of 620 nm.

### Cell proliferation studies using CyQUANT assay kit

Cell proliferation studies were also conducted using the CyQUANT cell proliferation kit (Life Technologies, Australia) which is a colorimetric based detection method for assessment of proliferating cells as the GR dye component binds directly to the DNA of proliferating cells. The cells were cultured at a cell density of 1 × 10^5^ cells/ml in a 96 well plate. The cells were allowed to grow at 37 °C at 5 % CO_2_ for 48 h. The plates were centrifuged at 1500 rpm and the media was discarded. Cells were treated for 24 h and all treatments were prepared in 1 % serum supplemented media. The incubation was done at 37 °C and 5 % CO_2_. Controls were maintained with only media and one group was incubated with 20 % serum supplemented media to achieve maximum proliferation acting as a positive control. After incubation for 24 h, the 96 well plates were centrifuged at 1200*g* for 5 min. The supernatant was carefully discarded and the cells were then air-dried for 30 min. After drying the plate was covered in aluminium foil and stored at −80 °C. The plate was removed from −80 °C storage after 48 h and 200 µl of the standard reaction solution was added and fluorescence was measured at excitation/emission wavelength of 485/530 nm using a microplate reader.

### Nanoparticle internalization studies using confocal and flow cytometry

For internalization assessment, cells were cultured in six well plates at an estimated population of around 2 × 10^5^ cells/ml for 24 h. Cells were time-dependently treated with 2g/ml Rhodamine labelled 100 µg/ml nanocomplexes in 1 % FBS RPMI-1640 media. Nanocomplex Rhodamine (Sigma) labelling was performed using standard EDC/NHS coupling reaction. The nanocomplex treated cells were centrifuged at 1200 rpm for 5 min and the pellet was redispersed in 1 ml PBS. The cell populations were sorted for nanocomplex internalization using fluorescence assisted cell sorter and nanocomplex untreated cells were used as control cell population. Confocal microscopy was performed with cells cultured in six well plates at an estimated 2 × 10^5^ cells/ml population. The cell treatments including controls including untreated control were centrifuged (1200 rpm, 5 min). The resulting pallet was redispersed in 200 µl PBS and slides were prepared for confocal fluorescence microscopy using the CytoSpin™ cell centrifugation system (1600 rpm, 6 min). The cell layer on the slide was allowed to air dry for 1 h, treated with DAPI mounting media and a coverslip is placed on top avoiding air bubbles. The coverslip was sealed on top of slide and observed under confocal microscope.

### Ex vivo assessment for validation of MRI and CT contrast of Hsp-70 Lf-PEG-ZF nanocomplex

*Psammomys obesus* was used as a model for validation of Hsp-70 Lf-PEG-ZF nanocomplex MRI contrast agent, ex vivo. The rat colony was maintained at the animal housing facility, Deakin University (Geelong, Australia). Breeding pairs were fed a standard rodent laboratory diet (Barastoc^®^, Pakenham, Australia). The animals were weaned at 4 weeks of age and switched to a standard laboratory diet, from which 12 % of energy was derived from fats, 63 % from carbohydrates, and 25 % from proteins. Animals were housed in a temperature-controlled room (22 ± 1 °C) with a 12 h light–dark cycle (light 0600–1800 h). The experimental samples for MRI and CT scans were prepared with two different strategies: extracted aorta and heart nanocomplex injected agar phantom and direct nanocomplex injection in the animal aorta post-sacrifice. For ex vivo assessment, juvenile [6 months old (n = 5)] and adult [16 (n = 5) and 18 month old (n = 5)] animals were humanely sacrificed in a CO_2_ chamber. Blood clotting within the cardiovascular vessel was prevented by promptly exposing the aorta followed by PBS perfusion immediately post-sacrifice. Any damage or dislocation of internal organs was minimized while exposing the aorta to keep the original structure intact. Nanocomplex contrast agent injection was followed right after PBS perfusion at the iliac bifurcation to prevent any blood clot formation in the aorta. Equal concentration of blood was removed prior to PBS perfusion and contrast agent injection. Young 6 month (n = 5) and adult 16 month old animals (n = 5) served as contrast agent treatments (100 µg/ml Hsp-70 Lf-PEG-ZF) and the 18 month old adults (n = 5) served as a control for MRI/computed tomography (CT) assessment. Animal thorax was sealed back and imaging was performed with all animals after 4 h of treatment. Scans were performed in coronal, sagittal and transverse orientation in order to have a three dimensional observation assessment of the atherosclerosis plaque sites. Constant scan parameters were maintained at 6.25 ms gradient echo (TE) and 8000 ms repetition time (TR) with fat saturation for all scans performed.

Later, the thorax and abdomen were rinsed with 70 % ethanol, an incision was made using sterile scissors and toothed forceps and the skin was removed. The thorax was opened exposing heart, lungs and the abdomen and the organs were carefully dissected and removed. Electrocautery was performed at all branches to remove the entire aorta and heart, simultaneously sealing all the vessel branches. The aorta was dissected below the iliac bifurcation using sterile micro-dissecting scissors. Intact aortae and hearts were moved to a petridish containing phosphate buffered saline (PBS) and PBS perfusion was performed at the iliac bifurcation end to remove any blood. The peri-aortic fat was meticulously removed preventing any damage to the blood vessel. The nanocomplex suspension was removed after 6 h of treatment and perfused with PBS to remove any unbound nanocomplex from the luminal aortic region. Finally, the tissues were fixed at 4 °C using 4 % Paraformaldehyde and set in 1 % agar in a customized diamagnetic sample holder for nominal magnetic resonance response. Nanocomplex injected aortae and hearts agar phantom were observed with the MRI and X-ray CT scanner.

### Histological analysis of plaque, nanocomplex binding and biomarkers

In order to detect the sites of atherosclerotic lesion/fatty streaks detected by MRI/CT imaging, the vasculature was divided into four parts: heart, aortic arch, descending aorta and abdominal aorta. 10 µm thick longitudinal and axial tissue sections were obtained using a cryotome on poly-lysine coated microscopic slides. The sections were fixed with acetone at −20 °C for 15 min. Standard protocol was followed to stain the sections with haematoxylin and eosin (H&E), methyl green and oil red-O to visualise the structure of cells and lesions/streaks in the aortic intima. For H&E staining the tissue sections were stained with haemotoxylin for 8 min and washed with tap water. Counterstaining was done with eosin for 2 min and the tissue was washed again with tap water. The tissue was subjected to acid alcohol for 2 s and washed gently with tap water. The slides were rehydrated/dehydrated in 70, 90 and 100 % ethanol sequentially for 2 min each. The sections were allowed to air dry and mounting media was added followed by placement of a coverslip avoiding any bubble formation. Standard manufacturer’s staining protocol was followed to stain the tissue sections with Prussian blue iron stain and Pararosalinine cellular staining kit (Sigma Aldrich) to observe nanoparticle targeting and localization. Immunohistological analysis was performed with 3,3′-Diaminobenzidine (DAB) staining against macrophage-specific CD44, Vascular endothelial growth factor (VEGF) and stress protein Hsp-70 for atherosclerosis plaque characterization. For DAB staining, the tissues were surrounded with a hydrophobic barrier using a barrier pen. To quench endogenous peroxidase activity, samples were incubated with 1-3 drops of peroxidase blocking reagent (3 % H_2_O_2_ in water or methanol) for 5–15 min. The samples were rinsed and gently washed in wash buffer for 5 min. To reduce non-specific hydrophobic interactions between the primary antibodies and the tissue, the sections were blocked with 1–3 drops of 3 % bovine serum albumin for 15 min. The slides were drained and any excess blocking reagent removed before proceeding to the next steps. The samples were incubated with primary antibodies with incubation buffer at 37 °C for 1 h or overnight at 4 °C. Manufacturer’s recommendations were followed regarding working dilution for the primary antibody. The samples were washed 3 times with wash buffer for 5 min each, and drained, then incubated with secondary antibody at 37 °C for 1 h. DAB reagent was then added and incubated for 5–10 min. The nucleus was counterstained either with methyl green or haematoxylin. The tissues were fixed with mounting media and a coverslip was placed avoiding any bubbles. Finally, the slides were observed under light microscope and images were captured.

## Additional files

10.1186/s12951-016-0157-1 Determination of serum stability of the synthesized MRI/CT contrast agents. The serum stability was conducted in with 1 mg/ml solution of Hsp-70 Lf-PEG-ZF, Hsp-70 Lf-ZF and Hsp-70 Ch-Lf-ZF nanoparticles in 10 % BSA for a period of 7 days. Analysis was performed using DLS.
10.1186/s12951-016-0157-1 Determination of T1, T2 and CT contrast of the synthesized MRI/CT contrast agent. A slight enhancement in T1 contrast (A) was observed in Hsp-70 Lf-PEG-ZF nanoparticles when compared to commercial ferrite nanoparticles. (B) A significant increase in the T2 contrast was observed in Hsp-70 Lf-PEG-ZF nanoparticles when compared to commercial ferrite nanoparticles. (C) A slight enhancement in CT contrast was observed in Hsp-70 Lf-PEG-ZF nanoparticles when compared to commercial ferrite nanoparticles.
10.1186/s12951-016-0157-1 Concentration-dependent lactate dehydrogenase (LDH) release cell cytotoxicity and CyQUANT cell proliferation assays with THP-1 and Jurkat cells. The cells were incubated with Hsp-70 Lf-PEG-ZF (A), Hsp-70 Lf-ZF (B) and Hsp-70 Ch-Lf-ZF (C) nanocomplexes.
10.1186/s12951-016-0157-1 Heat map images of MRI scans and determination of enhancement in T2 contrast. (A) The heat map images of representative images from Fig. 3 are represented with encircled region of interests (ROI). (B) The enhancement in contrast intensity was calculated from 5 different images of each treatment using imgae J software and is represented in a graph.
10.1186/s12951-016-0157-1 Detailed images from Fig. 3b. More images of ex vivo hart and aorta T2 MRI sagittal scans revealed a higher accumulation of Hsp-70 Lf-PEG-ZF nanoparticles in adult mice (16 months) when compared to juvenile mice (6 months old).
10.1186/s12951-016-0157-1 Site specific localisation of Hsp-70 Lf-PEG-ZF nanoparticles. The Hsp-70 Lf-PEG-ZF nanoparticles were found to be specifically bound to the region around the plaque in the aorta in adult mice.
10.1186/s12951-016-0157-1 Diagram schematically representing the steps involved in the chemical synthesis of Hsp-70 Lf-PEG-ZF nanocomplex.
10.1186/s12951-016-0157-1 Diagram schematically depicting the formation and steps involved in Hsp-70 Lf-PEG-ZF nanocomplex preparation.

## Abbreviations

ApoE: apolipoprotein E
BET: Brauner-Emmett-Teller
CVD: cardiovascular diseases
CT: computed tomography
DLS: dynamic light scattering
EDC: 1-ethyl-3-(3-di-methylaminopropyl) carbodiimide
NHS: N-hydroxysulfosuccinimide
FDA: food and drug administration
H&E: haematoxylin and eosin
Hsp: heat shock protein
LDH: lactate dehydrogenase
MRI: magnetic resonance imaging
M_S_: saturation magnetization
PBS: phosphate buffer saline
PAGE: poly acryl amide gel electrophoresis
PEG: polyethylene glycol
*P. obesus*: *Psammomys obesus*
SQUID: semiconductor quantum interference device
SPIONs: superparamagnetic iron oxide nanoparticles
TEM: transmission electron microscopy
XRD: X-ray diffraction
ZF: zinc doped ferrite
