# Correction to: Engineered atherosclerosis-specific zinc ferrite nanocomplex-based MRI contrast agents

**DOI:** 10.1186/s12951-019-0443-9

**Published:** 2019-02-28

**Authors:** Rajneesh Chaudhary, Kislay Roy, Rupinder Kaur Kanwar, Ken Walder, Jagat Rakesh Kanwar

**Affiliations:** School of Medicine, Faculty of Health, Centre for Molecular and Medical Research, Deakin University, Locked Bag 20000, Geelong, Victoria 3220 Australia

## Correction to: J Nanobiotechnol (2016) 14:6 10.1186/s12951-016-0157-1

After publication of the original article [1], the authors found that Fig. 2e (Hsp70-Ch-Lf-ZF in Jurkat cells, 2 h), Fig. 5b (control and HSP70-Lf-ZF) and Fig. 5c (control) contained incorrect images. This does not affect the figure legends, results and conclusions of the article.

**Fig. 2:**
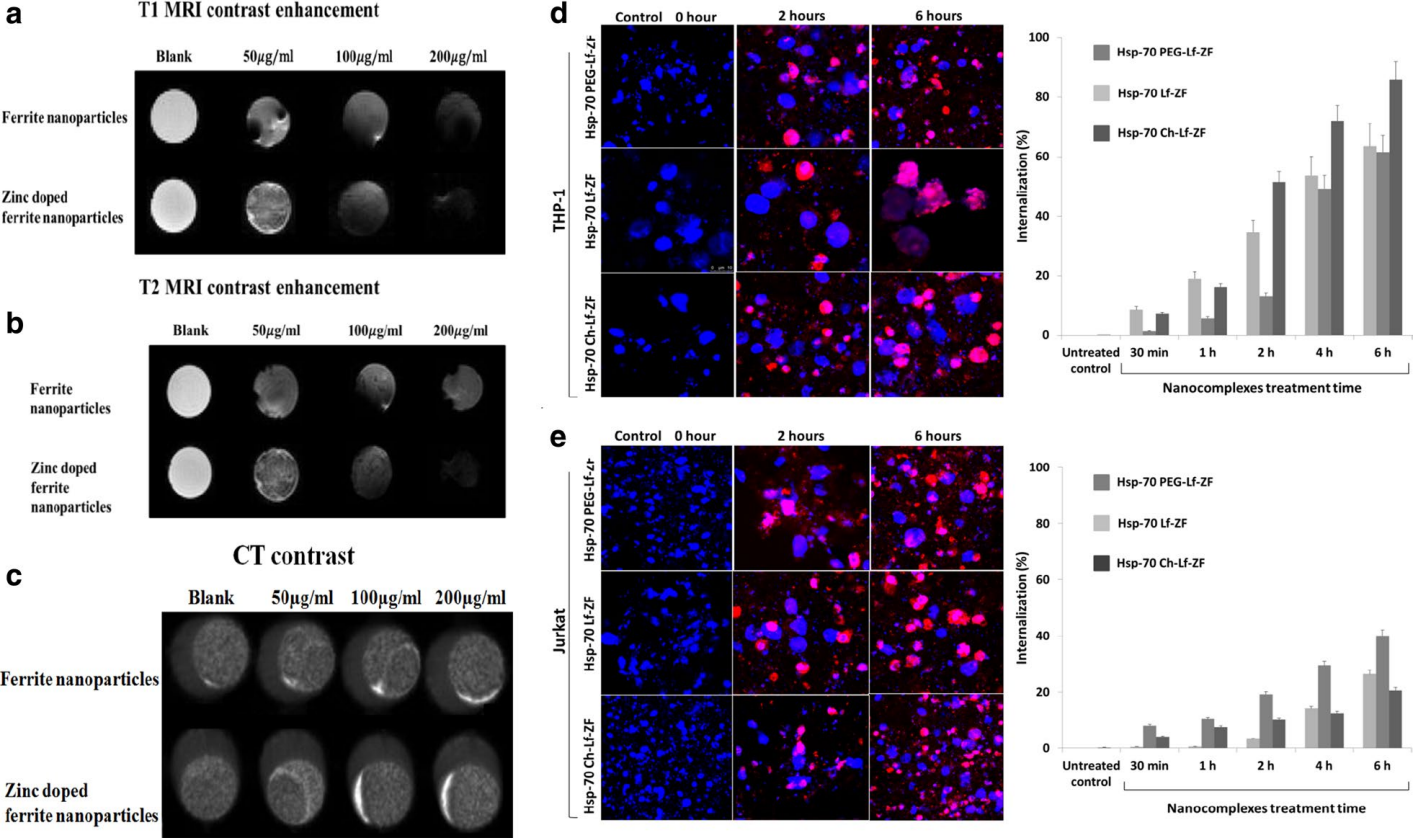
Enhancement in MRI/CT contrast and internalisation efficacy of nanoparticles. **a** Slight enhancement of T1 MRI contrast was observed in Hsp-70 Lf-PEG-ZF compared to ferrite nanoparticles. **b** Significant enhancement of T2 MRI contrast was observed in Hsp-70 Lf-PEG-ZF compared to ferrite nanoparticles. **c** Slight enhancement of CT contrast was observed in Hsp-70 Lf-PEG-ZF compared to ferrite nanoparticles. **d** Confocal microscopy internalization images and cell cytometric internalization assessment displaying time-dependent internalization of Hsp-70 Lf-ZF, Hsp-70 Ch-Lf-ZF and Hsp-70 Lf-PEG-ZF nanocomplexes in THP-1 cells and **e** Jurkat cells. Effective internalization was observed at 2 h Hsp-70 Lf-ZF nanocomplex post-treatment

**Fig. 5:**
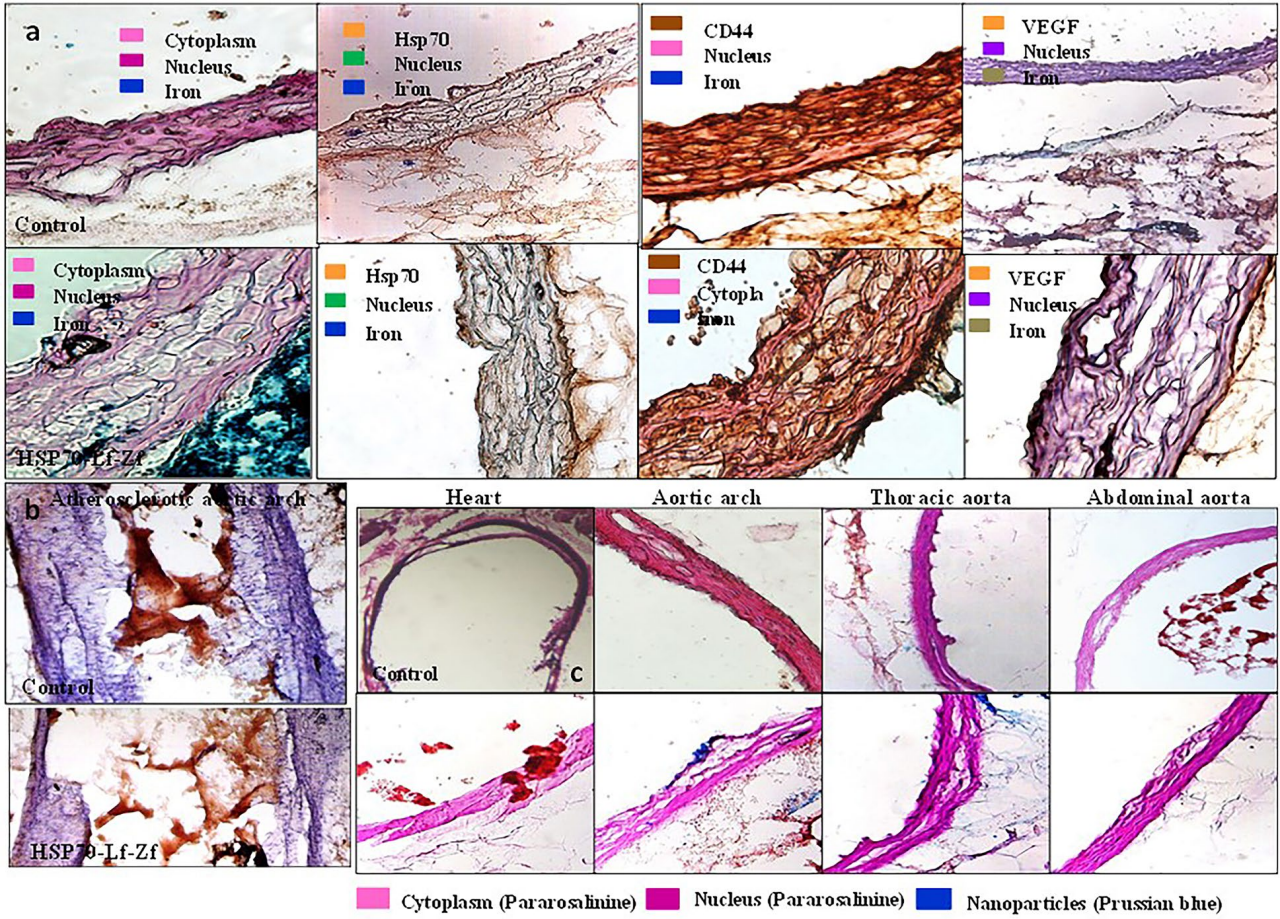
Histological assessment images for nanoparticle distribution and vascular morphology assessment of intra-aortic Hsp-70 Lf-PEG-ZF nanocomplex contrast agent injected *P. obesus* aorta and heart sections. Nanoparticle distribution images of axially sectioned captured at ×20 magnification and the tissues were and stained with Prussian blue iron stain and Pararosaniline cellular and nuclear stain. **a** Immunohistological images of axially sectioned aortic arch from control rats and Hsp-70 Lf-PEG-ZF injected rats, stained against Prussian blue iron stain, Hsp-70, CD44 and VEGF respectively captured at ×20 magnification. **b** Longitudinal section of aortic arch tissue arch from control rats and Hsp-70 Lf-PEG-ZF injected rats stained against hematoxylin nuclear and oil red-O lipid stain captured at ×10 magnification. Advanced atherosclerotic plaque causing severe luminal stenosis was observed at the aortic arch region. Pathological intimal thickening with significant lipid deposition within the intima, media, necrotic core as well as extracellular lipid accumulation in the aortic lumen was observed. **c** Nanoparticle accumulation could not be observed in the heart with no disruption of the pericardium. Aortic arch section displayed severely disrupted intimal structural integrity and widening of first interlamellar spaces with atherosclerotic micro lesions. The nanoparticles can be seen attached with the intimal endothelium, within the intima and across into the adventitia. Descending thoracic aorta with complex micro lesions displayed significant nanoparticle accumulation within the intima and adventitia. Intima widening could be observed with *blue steaks* in the intima indicating cellular internalization and interlamellar contrast agent accumulation. The iliac bifurcation section adjacent to the abdominal aorta with partly disrupted intimal integrity and fewer lesions was observed. The endothelial layer was mostly conserved with minimal endothelium denudation. Adventitial nanoparticulate contrast agent accumulation was also minimal abdominal aortic region. Number of micro-atherosclerotic lesions and intimal widening was significantly less in comparison to aortic arch and descending thoracic aorta. Hematoxylin, methyl green and pararosaniline were used for nuclear staining and antibodies were stained with 3,3′-diaminobenzidine DAB

The correct versions of Figs. 2 and 5 are included with this correction.

The address of the authors was revised and is given in this Correction.
