# Supplementary material for: Engineered atherosclerosis-specific zinc ferrite nanocomplex-based MRI contrast agents
Source: J Nanobiotechnology. 2016 Jan 16;14:6. doi: 10.1186/s12951-016-0157-1 (PMC4715323; doi:10.1186/s12951-016-0157-1)
Supplement: Supplementary file 1 — 10.1186/s12951-016-0157-1 Determination of serum stability of the synthesized MRI/CT contrast agents. The serum stability was conducted in with 1 mg/ml solution of Hsp-70 Lf-PEG-ZF, Hsp-70 Lf-ZF and Hsp-70 Ch-Lf-ZF nanoparticles in 10 % BSA for a period of 7 days. Analysis was performed using DLS. [file 12951_2016_157_MOESM1_ESM.docx]

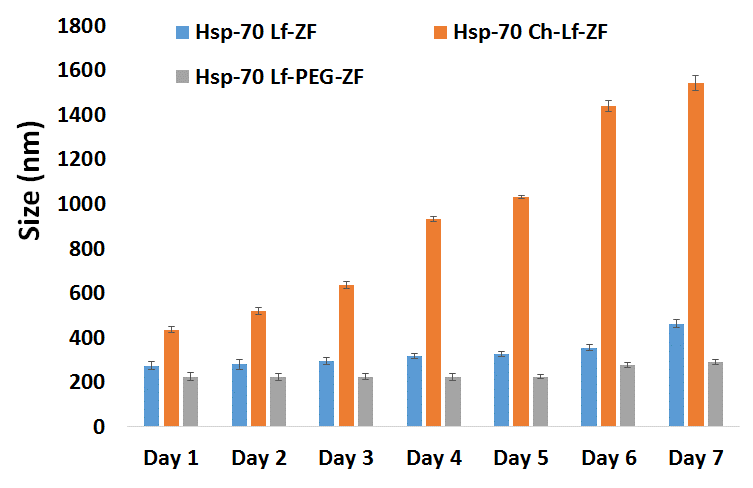


**Figure S1. Determination of serum stability of the synthesized MRI/CT contrast agents.** The serum stability was conducted in with 1mg/ml solution of Hsp-70 Lf-PEG-ZF, Hsp-70 Lf-ZF and Hsp-70 Ch-Lf-ZF nanoparticles in 10% BSA for a period of 7 days. Analysis was performed using DLS.
