# Supplementary material for: Engineered atherosclerosis-specific zinc ferrite nanocomplex-based MRI contrast agents
Source: J Nanobiotechnology. 2016 Jan 16;14:6. doi: 10.1186/s12951-016-0157-1 (PMC4715323; doi:10.1186/s12951-016-0157-1)
Supplement: Supplementary file 2 — 10.1186/s12951-016-0157-1 Determination of T1, T2 and CT contrast of the synthesized MRI/CT contrast agent. A slight enhancement in T1 contrast (A) was observed in Hsp-70 Lf-PEG-ZF nanoparticles when compared to commercial ferrite nanoparticles. (B) A significant increase in the T2 contrast was observed in Hsp-70 Lf-PEG-ZF nanoparticles when compared to commercial ferrite nanoparticles. (C) A slight enhancement in CT contrast was observed in Hsp-70 Lf-PEG-ZF nanoparticles when compared to commercial ferrite nanoparticles. [file 12951_2016_157_MOESM2_ESM.docx]

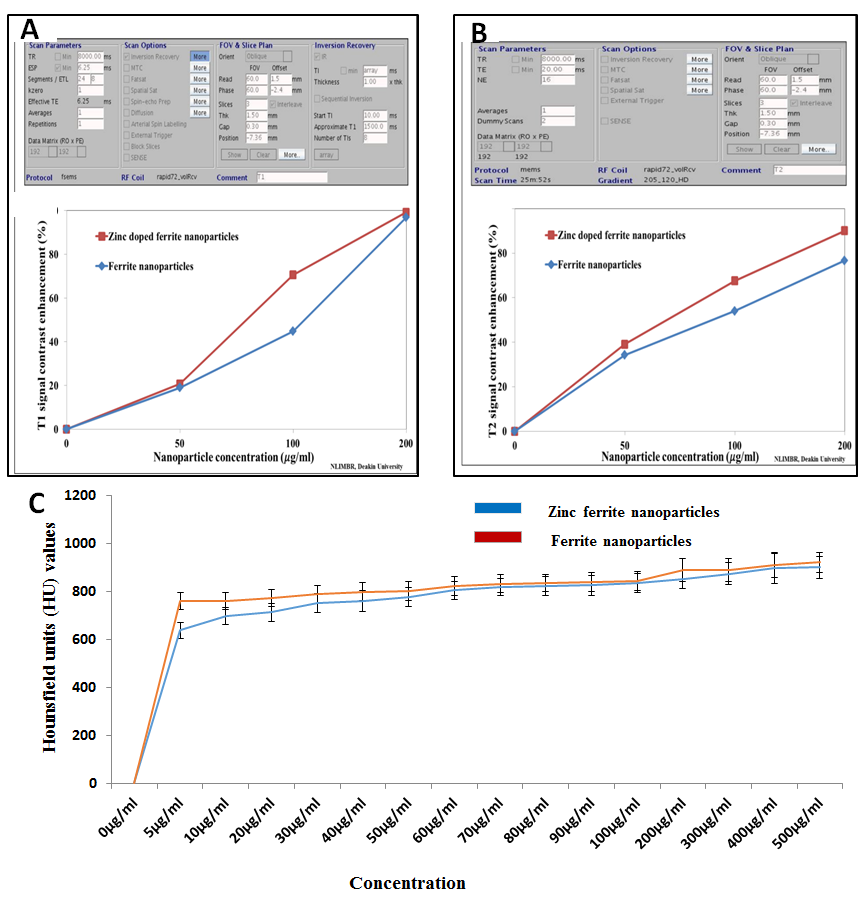


**Figure S2. Determination of T1, T2 and CT contrast of the synthesized MRI/CT contrast agent.** A slight enhancement in T1 contrast (A) was observed in Hsp-70 Lf-PEG-ZF nanoparticles when compared to commercial ferrite nanoparticles. (B) A significant increase in the T2 contrast was observed in Hsp-70 Lf-PEG-ZF nanoparticles when compared to commercial ferrite nanoparticles. (C) A slight enhancement in CT contrast was observed in Hsp-70 Lf-PEG-ZF nanoparticles when compared to commercial ferrite nanoparticles.
