# Supplementary material for: Engineered atherosclerosis-specific zinc ferrite nanocomplex-based MRI contrast agents
Source: J Nanobiotechnology. 2016 Jan 16;14:6. doi: 10.1186/s12951-016-0157-1 (PMC4715323; doi:10.1186/s12951-016-0157-1)
Supplement: Supplementary file 3 — 10.1186/s12951-016-0157-1 Concentration-dependent lactate dehydrogenase (LDH) release cell cytotoxicity and CyQUANT cell proliferation assays with THP-1 and Jurkat cells. The cells were incubated with Hsp-70 Lf-PEG-ZF (A), Hsp-70 Lf-ZF (B) and Hsp-70 Ch-Lf-ZF (C) nanocomplexes. [file 12951_2016_157_MOESM3_ESM.docx]

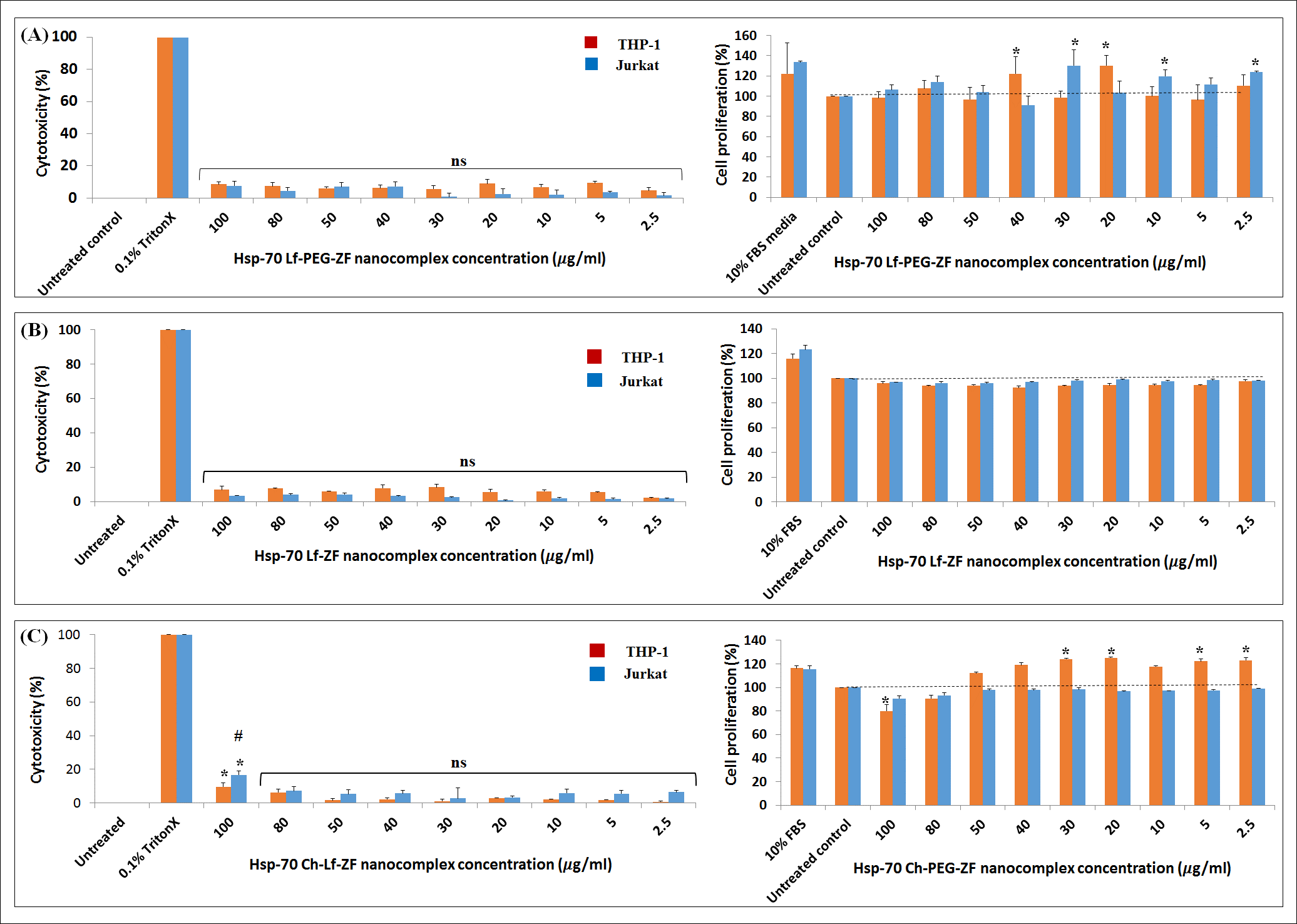


**Figure S3. Concentration-dependent lactate dehydrogenase (LDH) release cell cytotoxicity and CyQUANT cell proliferation assays with THP-1 and Jurkat cells.** The cells were incubated with Hsp-70 Lf-PEG-ZF (A), Hsp-70 Lf-ZF (B) and Hsp-70 Ch-Lf-ZF (C) nanocomplexes.
