# Supplementary material for: Engineered atherosclerosis-specific zinc ferrite nanocomplex-based MRI contrast agents
Source: J Nanobiotechnology. 2016 Jan 16;14:6. doi: 10.1186/s12951-016-0157-1 (PMC4715323; doi:10.1186/s12951-016-0157-1)
Supplement: Supplementary file 4 — 10.1186/s12951-016-0157-1 Heat map images of MRI scans and determination of enhancement in T2 contrast. (A) The heat map images of representative images from Fig. 3 are represented with encircled region of interests (ROI). (B) The enhancement in contrast intensity was calculated from 5 different images of each treatment using imgae J software and is represented in a graph. [file 12951_2016_157_MOESM4_ESM.docx]

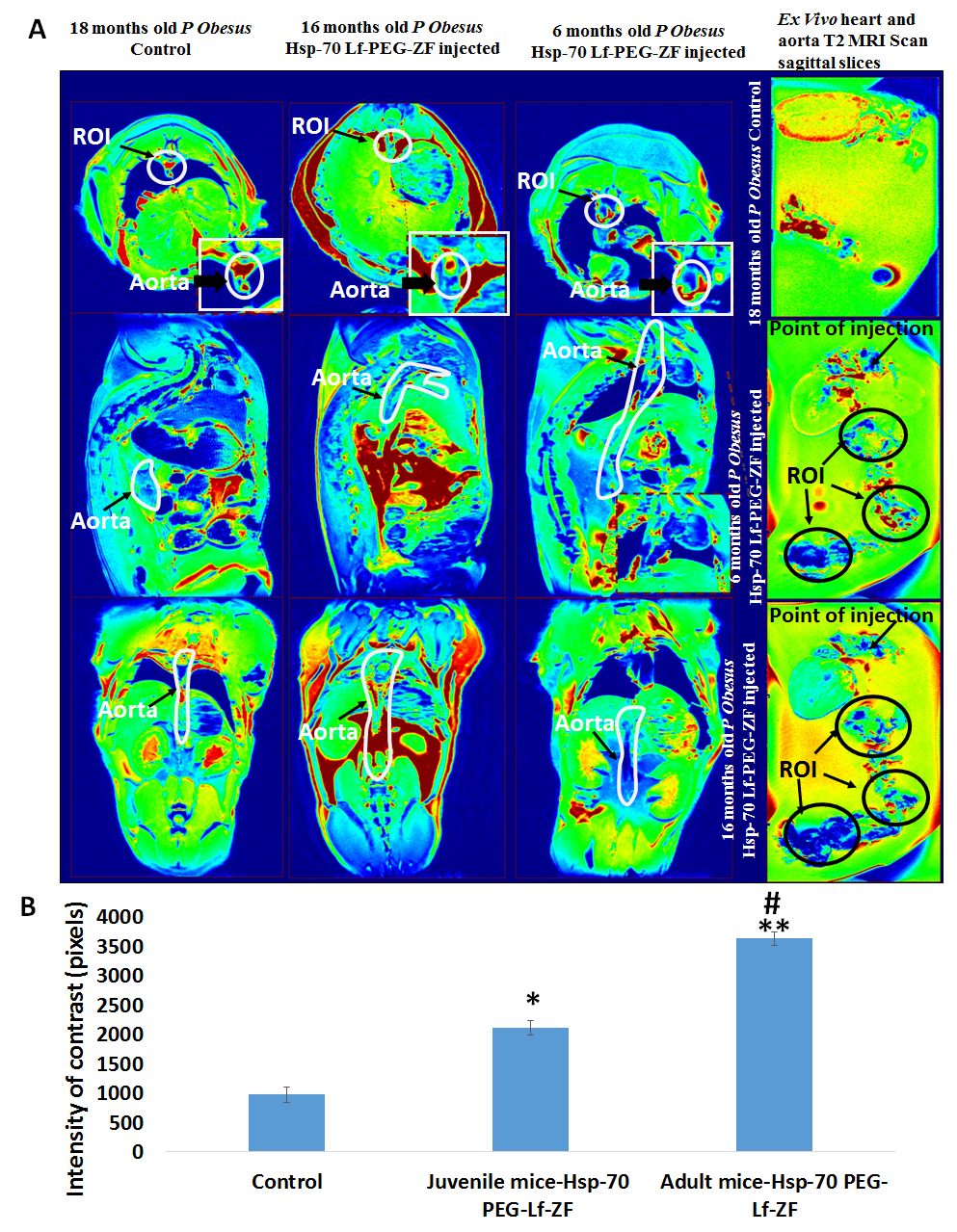


**Figure S4. Heat map images of MRI scans and determination of enhancement in T2 contrast.** (A) The heat map images of representative images from figure 3 are represented with encircled region of interests (ROI). (B) The enhancement in contrast intensity was calculated from 5 different images of each treatment using imgae J software and is represented in a graph.
