# Supplementary material for: Engineered atherosclerosis-specific zinc ferrite nanocomplex-based MRI contrast agents
Source: J Nanobiotechnology. 2016 Jan 16;14:6. doi: 10.1186/s12951-016-0157-1 (PMC4715323; doi:10.1186/s12951-016-0157-1)
Supplement: Supplementary file 5 — 10.1186/s12951-016-0157-1 Detailed images from Fig. 3b. More images of ex vivo hart and aorta T2 MRI sagittal scans revealed a higher accumulation of Hsp-70 Lf-PEG-ZF nanoparticles in adult mice (16 months) when compared to juvenile mice (6 months old). [file 12951_2016_157_MOESM5_ESM.docx]

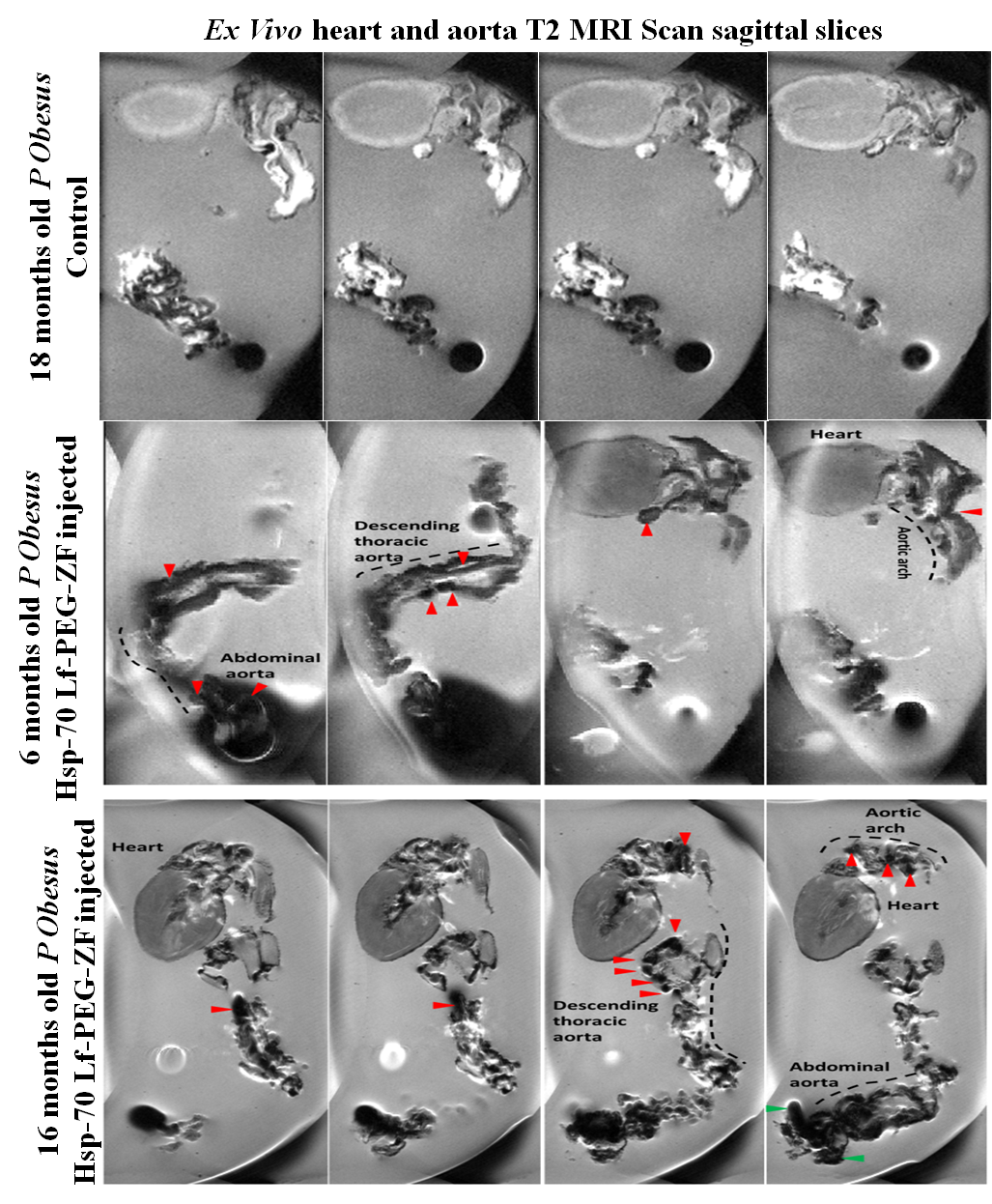


**Figure S5. Detailed images from Figure 3B.** More images of ex vivo hart and aorta T2 MRI sagittal scans revealed a higher accumulation of Hsp-70 Lf-PEG-ZF nanoparticles in adult mice (16 months) when compared to juvenile mice (6 months old).
