# Supplementary material for: Engineered atherosclerosis-specific zinc ferrite nanocomplex-based MRI contrast agents
Source: J Nanobiotechnology. 2016 Jan 16;14:6. doi: 10.1186/s12951-016-0157-1 (PMC4715323; doi:10.1186/s12951-016-0157-1)
Supplement: Supplementary file 6 — 10.1186/s12951-016-0157-1 Site specific localisation of Hsp-70 Lf-PEG-ZF nanoparticles. The Hsp-70 Lf-PEG-ZF nanoparticles were found to be specifically bound to the region around the plaque in the aorta in adult mice. [file 12951_2016_157_MOESM6_ESM.docx]

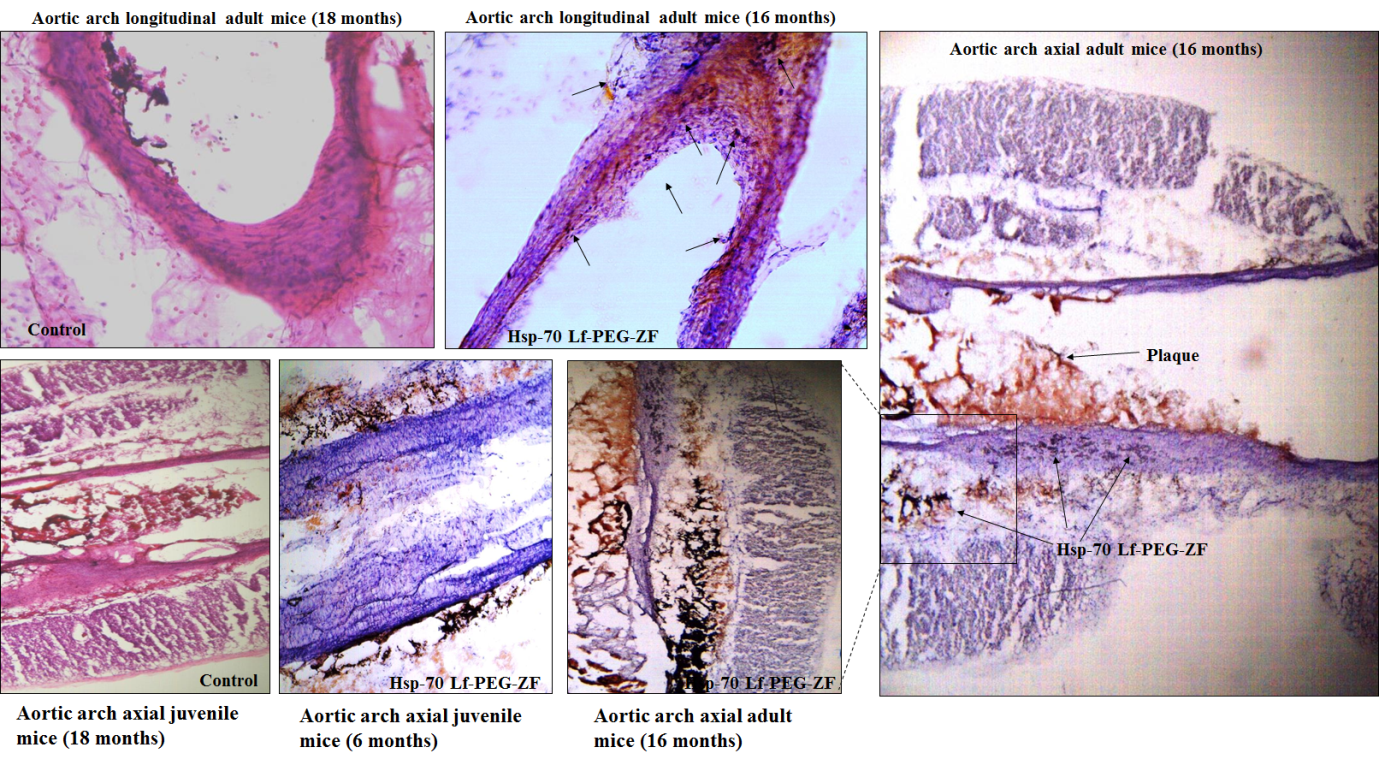


**Figure S6. Site specific localisation of Hsp-70 Lf-PEG-ZF nanoparticles.** The Hsp-70 Lf-PEG-ZF nanoparticles were found to be specifically bound to the region around the plaque in the aorta in adult mice.
