# Supplementary material for: Engineered atherosclerosis-specific zinc ferrite nanocomplex-based MRI contrast agents
Source: J Nanobiotechnology. 2016 Jan 16;14:6. doi: 10.1186/s12951-016-0157-1 (PMC4715323; doi:10.1186/s12951-016-0157-1)
Supplement: Supplementary file 7 — 10.1186/s12951-016-0157-1 Diagram schematically representing the steps involved in the chemical synthesis of Hsp-70 Lf-PEG-ZF nanocomplex. [file 12951_2016_157_MOESM7_ESM.docx]

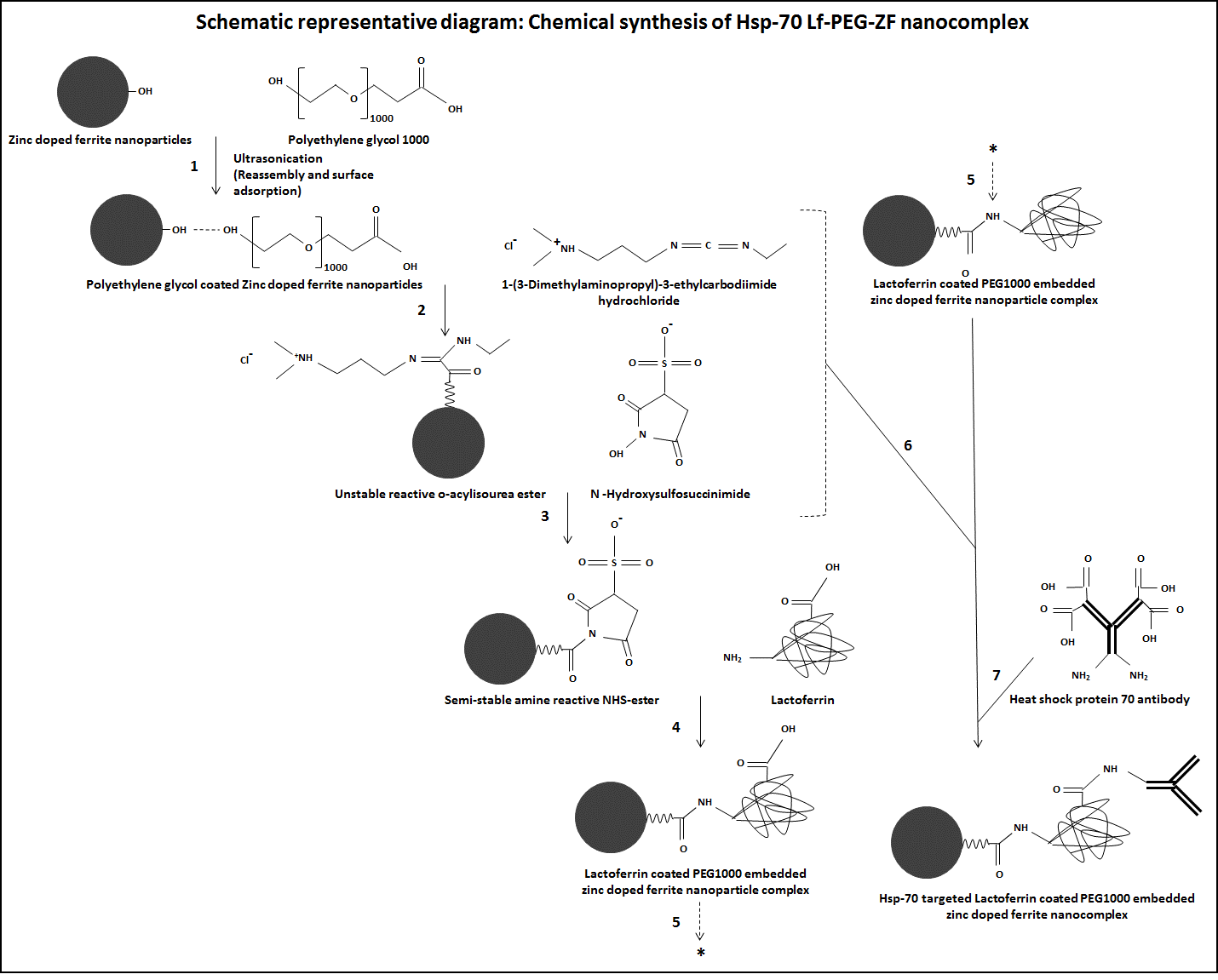


**Figure S7.** Diagram schematically representing the steps involved in the chemical synthesis of Hsp-70 Lf-PEG-ZF nanocomplex.
