# Supplementary material for: Engineered atherosclerosis-specific zinc ferrite nanocomplex-based MRI contrast agents
Source: J Nanobiotechnology. 2016 Jan 16;14:6. doi: 10.1186/s12951-016-0157-1 (PMC4715323; doi:10.1186/s12951-016-0157-1)
Supplement: Supplementary file 8 — 10.1186/s12951-016-0157-1 Diagram schematically depicting the formation and steps involved in Hsp-70 Lf-PEG-ZF nanocomplex preparation. [file 12951_2016_157_MOESM8_ESM.docx]

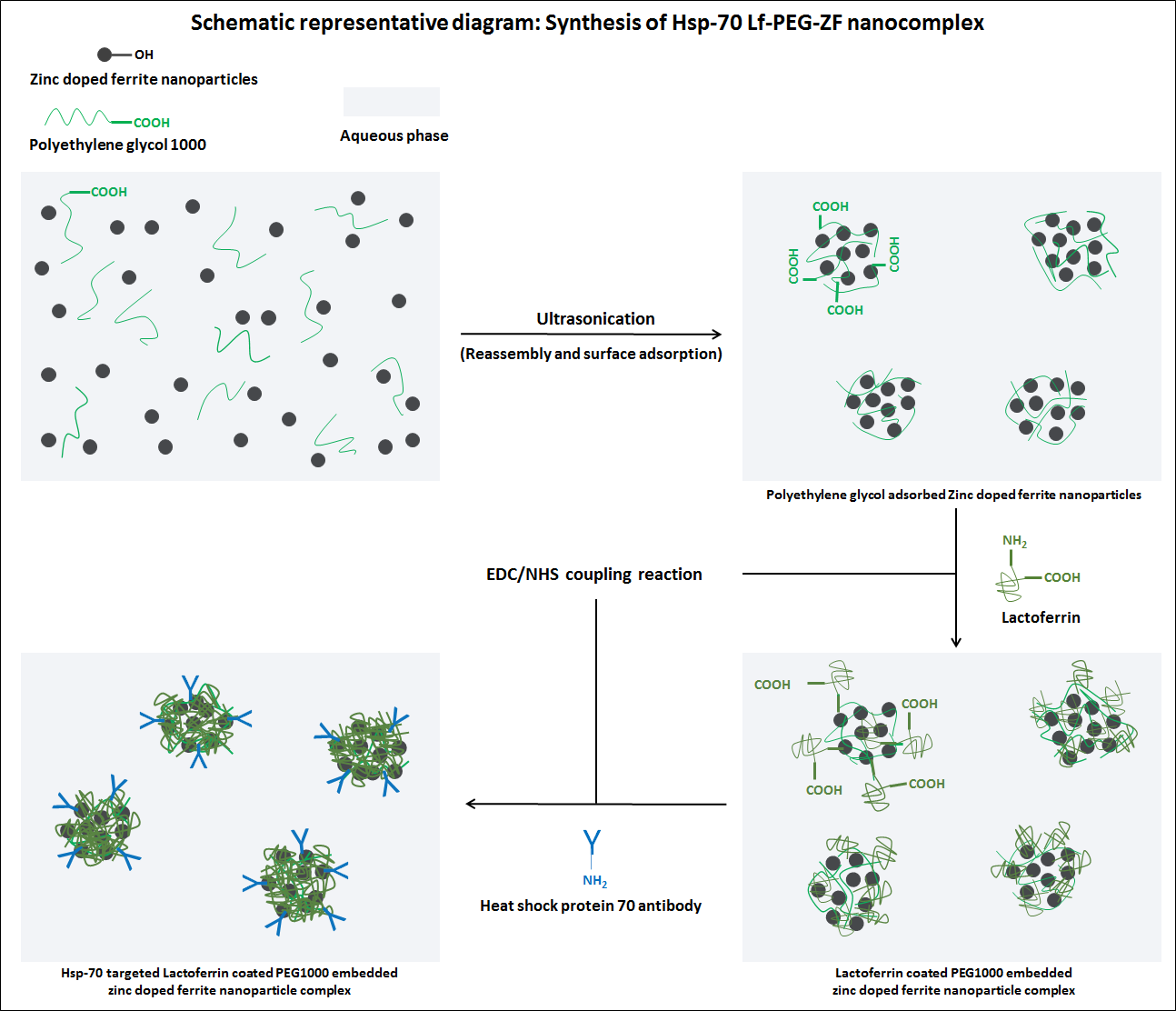


**Figure S8.** Diagram schematically depicting the formation and steps involved in Hsp-70 Lf-PEG-ZF nanocomplex preparation.
